# The influence of HSP inducers on salinity stress in sterlet sturgeon (*Acipenser ruthenus*): *In vitro* study on HSP expression, immune responses, and antioxidant capacity

**DOI:** 10.1016/j.cstres.2024.06.004

**Published:** 2024-06-22

**Authors:** Sevda Zarei, Hossein Ghafouri, Leila Vahdatiraad, Behrooz Heidari

**Affiliations:** 1 Department of Biology, Faculty of Science, University of Guilan, Rasht, Iran; 2 Department of Marine Sciences, The Caspian Sea Basin Research Center, University of Guilan, Rasht, Iran

**Keywords:** *Acipenser ruthenus*, Heat shock protein, HSPi, Salinity, Stress response

## Abstract

Heat shock proteins (HSPs) play a crucial role in antioxidant systems, immune responses, and enzyme activation during stress conditions. Salinity changes can cause stress and energy expenditure in fish, resulting in mortality, especially in fingerlings. The purpose of this study was to examine the relationship between salinity and HSPs in stressed fish by assessing the effects of various HSP inducers (HSPis), including Pro-Tex® (800 mM), amygdalin (80 mM), and a novel synthetic compound derived from pirano piranazole (80 µM), on isolated cells from Sterlet Sturgeon (*Acipenser ruthenus*) exposed to 13 ‰ salinity (S13). After liver, kidney, and gill cells were cultured, the HSPi compounds were treated *in vitro* in the presence and absence of salinity. The expression patterns of HSP27, HSP70, and HSP90 were assessed by Western blotting. Biochemical enzymes (aspartate aminotransferase, alanine aminotransferase, alkaline phosphatase, and lactate dehydrogenase), cortisol levels, and immune parameters (component 3, immunoglobulin M, and lysozyme) were measured before and after treatment with HSPis and HSPi + S13. According to these findings, HSPis positively modulate HSP expression, immune responses, and antioxidant levels. Furthermore, they increased *in vitro* cell survival by maintaining cortisol levels and biochemical enzyme activities in *A. ruthenus* under saline conditions (*P* < 0.0001). In conclusion, HSPis can increase *A. ruthenus* resistance to salinity stress. However, the results also indicated that these compounds can reverse the adverse effects of salinity. The effectiveness of this approach depends on further research into the effects of these ecological factors on the health status of the species, especially *in vivo* and in combination with other stresses.

## Highlights

• Heat shock proteins (HSPs) play important roles in the immune and antioxidant systems.
• Sterlet sturgeon cells were treated with HSP inducers (HSPi) in the presence or absence of salinity.
• A significant increase in HSP expression was found after HSPi and salinity stress.
• HSPi can be a powerful modulator of the immune response and survival in *A*. *ruthenus* under salinity.

## Graphical abstract

**Figure:**
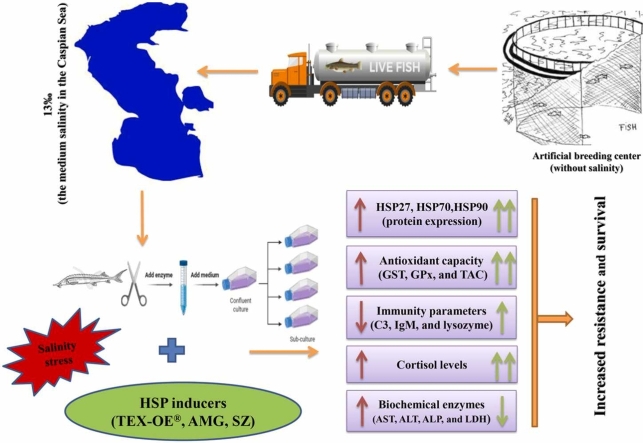

## Introduction

Iranian sturgeons live mostly on the bottoms of beaches in the central and southern Caspian Sea.^1^, ^2^ Several factors contribute to the plight of Caspian sturgeon populations, including overfishing, water pollution, changes in water chemistry, and blocked migration routes. As a result, approximately 85 % of sturgeons are threatened.^1^, ^3^ In recent years, due to the conditions leading to extinction, sturgeons have been cultivated artificially due to their value in the Caspian Sea^4^. In Guilan Province, the Sefidroud River flows into the Caspian Sea and hosts one of the best fish release stations. Its salinity ranges from 2 to 12 parts per trillion (ppt) in the middle, upstream, and estuary sections.^3^, ^5^, ^6^ The salts in the Caspian Sea are relatively salty. However, this amount is less than that in many other seas and oceans, so on average, there are 12–13 g of salt per liter of water.^7^, ^8^ Fish fully adapt to their environment by gradually increasing the salt content of the water. Upstream or in the middle of the river, which has low salinity, the fish fry should be left, and when they reach the mouth of the river and the Caspian Sea, which has high salinity, they should gradually adapt to the environment.^9^, ^10^ Fry fish are released immediately near the mouth or entrance of the Caspian Sea to avoid being hunted by humans and other animals. Because of their sudden release at high salinity, fish lose the opportunity to compromise and complete their osmotic system, and a high shock occurs.^11^

The most common physiological stress factor is water salinity, which disrupts physiological functions and stimulates stress responses.^12^, ^13^ With respect to global changes, salinity has received less attention than other environmental factors, such as temperature and carbonate chemistry.^14^ Due to climate change, salinity variations were expected to increase, which can lead to stress and ultimately reduce fish production.^15^ Especially for larvae and fingerlings, temperature and salinity influence growth, development, and survival.^16^

In the absence of adequate protection from antioxidant barrier, an imbalance in oxidative system can cause oxidative stress, leading to cell damage and pathology.^17^, ^18^ Several studies have shown that oxidative stress and salinity changes are related in fish.^9^, ^13^, ^19^ The antioxidant enzymes glutathione S-transferase (GST), glutathione peroxidase (GPx), and total antioxidant capacity (TAC) can protect cells from oxidative stress.^20^, ^21^, ^22^ As a result, biomarkers of oxidative stress can be used to assess fish physiology. Additionally, environmental and geographical variations influence hematological and biochemical parameters.^23^, ^24^ During stress, cortisol is the main corticosteroid that regulates water balance.^25^ Stress is primarily measured by cortisol, which is an indicator of fish stress. Stress triggers physiological mechanisms to maintain body homeostasis, which can affect fish immunity.^26^ The innate immune system of fish includes components such as complement component 3 (C3), lysozyme (LYZ), and immunoglobulin M (IgM), which respond to pathogens and environmental stresses.^27^ For example, fish C3 levels are affected by environmental stress.^28^

Different stress reactions occur at the cellular level in fish in response to stressors. An important mechanism for protecting cells from various stressors is the synthesis of heat shock proteins (HSPs).^29^, ^30^ In addition to maintaining cellular integrity during normal development, HSPs play an important role in physiopathology.^31^ Fish tissues, cell lines, and primary cultures of various types of cells expressing HSPs have been described. Data suggested that HSP expression was influenced by a variety of biological and abiotic stressors, including infectious pathogens, heat, cold shock, salinity, and environmental contaminants.^32^, ^33^ Additionally, environmental factors and oxidative stress were shown to upregulate the protein levels of HSP27, HSP70, and HSP90.^34^, ^35^, ^36^ HSP70 also plays an important role in fish adaptation to salinity changes.^9^, ^37^ There are compounds found in nature or manufactured that can stimulate HSP expression. They can activate HSPs and thereby induce HSPs.^38^ Among these compounds is Pro-Tex®, a resistant precursor that induces HSPs in organisms such as Persian sturgeons.^30^ Pro-Tex® is the soluble form of TEX-OE®, a substance derived from *Opuntia ficus indica* or Nopal cactus (NOP).^4^ Amygdalin (AMG) is another HSP inducer (HSPi) that is frequently used.^39^ According to several studies, pirano-piranazole-based compounds upregulate the expression of HSP70 by activating the heat shock factor-1 (HSF1) gene.^40^, ^41^

Toxicology, carcinogenesis, and gene regulation and expression can all be studied using continuous cell lines.^42^ The acid–base balance is regulated by gill cells, whereas metabolic regulation and detoxification are regulated by liver cells.^43^, ^44^ In addition, adverse environmental conditions can also adversely affect the kidneys.^45^ Cell-based *in vitro* models mimic the structure, absorption, detoxification, and other pathophysiological characteristics of fish liver, gills, and kidneys. By providing a high-throughput platform, toxicity screening can be accelerated. In this study, we aimed to increase the survival of cells extracted from sterlet sturgeon tissues (liver, gills, and kidney) under salinity stress. We studied the effects of two commercial HSPis, Tex-OE, and AMG, as well as a newly synthesized pirano-piranazole-based inducer called synthesis compound, on HSP expression patterns, antioxidant parameters, biochemical enzyme activity, immune responses, and cortisol levels.

## Results

### Cell viability (%)

The levels of cell survival for the chosen dose treatments differed among liver, gill, and kidney cells (Figure 2). Compared to the control (100 %), S13 reduced cell viability in the treated cells of all 3 tissues (72.26 %, 67.95 %, and 70.11 %). Among all the HSPi compounds studied, N800 had the least viability in the liver, gills, and kidney (86.10 %, 86.61 %, and 82.88 %, respectively). Compared to the S13 group, the HSPi + S13 groups, including the SZ80 + S13 (in liver: 94.16 %, in gill: 91.80 %, and kidney: 92.93 %), A80 + S13 (in liver: 103.73 %, gill: 102.99 %, and kidney: 101.81 %) and N800 + S13 (liver: 99.06 %, gill: 100 %, and kidney: 99.66 %) groups, all exhibited significantly increased cell survival (*P <* 0.0001).

**Fig. 1:**
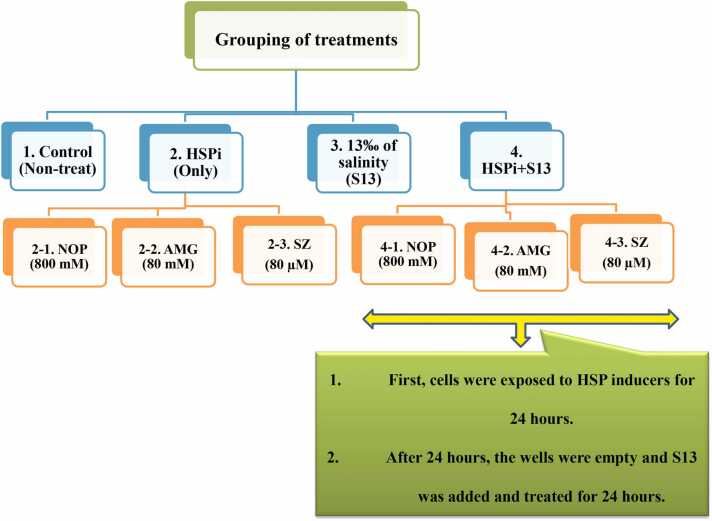
Grouping of treatments. Eight groups were considered: (1) control cells without treatment (Cr), HSPi groups containing (2) 80 mM amygdalin (A80), (3) 80 µM SZ (SZ80), (4) 800 mM Nopal endurance (N800), (5) 13 ‰ salinity stress group (medium salinity in the Caspian Sea; S13), and HSPi + salinity stress groups containing (HSPi + S13): (6) A80 + S13, (7) SZ80 + S13, and (8) N800 + S13. A total of 5 × 10^5^ cells/mL from each tissue (kidney, liver and gill) were counted and transferred to a 24-well plate. Each group was repeated 3 times, and after 24 h, the cells were collected from the wells and used for subsequent tests. Abbreviations used: AMG, amygdalin; HSP, heat shock protein; HSPi, HSP inducer; NOP, Nopal cactus.

**Fig. 2:**
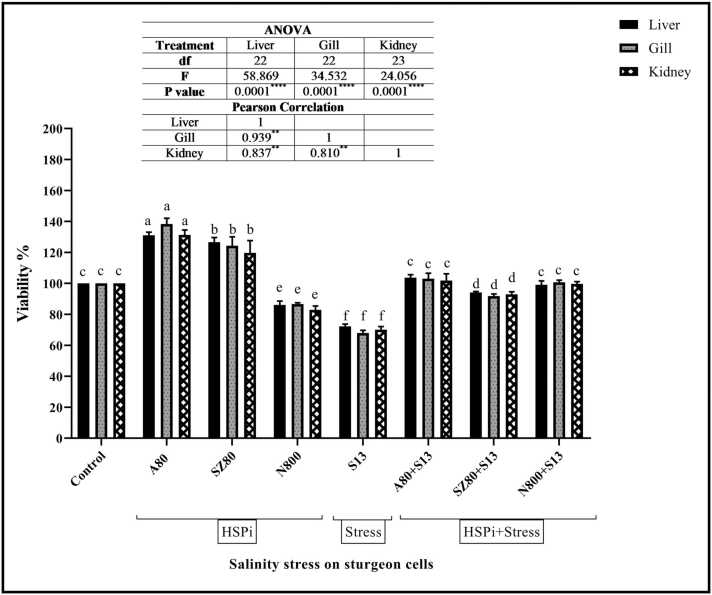
The cell survival rate (%) of cells isolated from *Acipenser ruthenus* tissues (mean ± SEM) undertreated with selective doses and salinity stress**.** A dose of 800 mM NOP (N800), 80 mM AMG (A80) and 80 µM SZ (SZ80) was selected as the optimal dose of HSPi. Additionally, a 13 ‰ salinity stress group (medium salinity in the Caspian Sea; S13) was selected for the test. In eight treatment groups, each parameter was measured three times. Within each column, different letters indicate significantly different groups according to Duncan's test (a, b, c, and.; *P* < 0.0001). Positive correlations were observed between all three cell lines. Abbreviations used: AMG, amygdalin; ANOVA, analysis of variance; HSPi, heat shock protein inducer; NOP, Nopal cactus.

### HSP response to salinity stress

#### HSP27

**Fig. 3:**
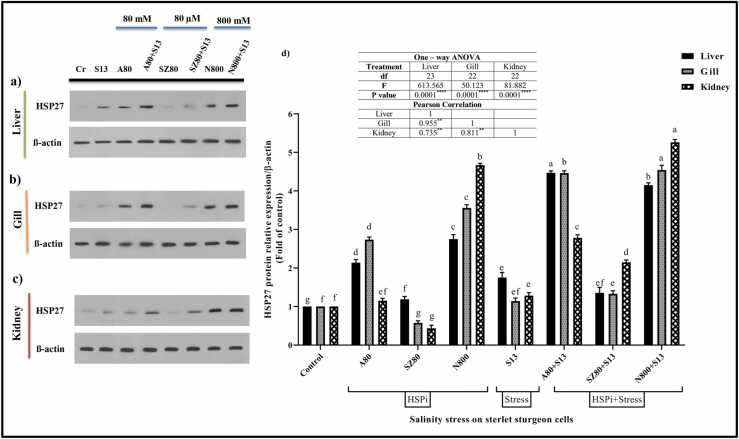
HSP27 relative expression analysis through western blotting. (a) Liver, (b) gill and (c) kidney cells. (d) Eight groups were considered: (1) control cells without treatment (Cr), HSPi groups containing (2) 80 mM amygdalin (A80), (3) 80 µM SZ (SZ80), (4) 800 mM Nopal endurance (N800), (5) 13 ‰ salinity stress group (medium salinity in the Caspian Sea; S13), and HSPi + salinity stress groups containing (HSPi + S13): (6) A80 + S13, (7) SZ80 + S13, and (8) N800 + S13. There were eight treatment groups in which each parameter was measured three times. HSP27 protein levels are shown as fold of control values (means ± SEMs), and within each column, different letters indicate significantly different groups according to Duncan's test (a, b, c, and… *P* < 0.0001). All three cell lines exhibited a positive correlation. Abbreviations used: ANOVA, analysis of variance; HSP, heat shock protein; HSPi, HSP inducer.

HSP27 protein expression varied significantly among liver, kidney, and gill cells (Figure 3; *P* < 0.0001). Compared to the control group, all treatment groups showed increased HSP27 expression in liver cells (Figure 3(a) and (d); *P* < 0.0001). Among all the HSPi compounds, SZ80 expressed the lowest amount of protein (1.188-fold). Among the groups, A80 + S13 (4.469-fold) and N800 + S13 (4.151-fold) had the highest HSP27 expression (*P* < 0.0001). In gill cells, the S13 group (1.141-fold) had a lower effect on HSP27 expression than the control group. Treatment with N800 + S13 also resulted in maximal HSP27 expression (4.542-fold; *P* < 0.0001). Like in liver cells, among all HSPi compounds, SZ80 exhibited the lowest protein expression level (0.575-fold) in gill cells (Figure 3(b) and (d); *P* < 0.0001). Treatment with SZ80 (0.435-fold) and N800 + S13 (5.262-fold) resulted in fewer or more HSP27, respectively, in kidney cells than in control cells. The HSP27 protein expression in the S13 group (1.283-fold) was slightly greater than that in the control group. Cells treated with HSPi compounds followed by salinity stress (S13) showed increased protein expression (HSPi + S13; *P* < 0.0001; Figure 3(c) and (d)).

#### HSP70

**Fig. 4:**
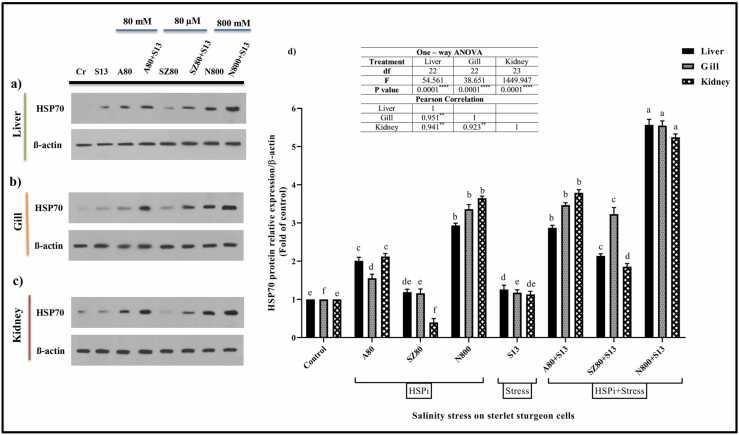
Analyzing HSP70 relative expression using western blots. (a) Liver, (b) gill and (c) kidney cells. (d) Eight groups were considered: (1) control cells without treatment (Cr), HSPi groups containing (2) 80 mM amygdalin (A80), (3) 80 µM SZ (SZ80), (4) 800 mM Nopal endurance (N800), (5) 13 ‰ salinity stress group (medium salinity in the Caspian Sea; S13), and HSPi + salinity stress groups containing (HSPi + S13): (6) A80 + S13, 7) SZ80 + S13, and (8) N800 + S13. There were eight treatment groups in which each parameter was measured three times. HSP70 protein levels are shown as fold of control values (mean ± SEM), and within each column, different letters indicate significantly different groups according to Duncan's test (a, b, c, and… *P* < 0.0001). There was a positive correlation between all three cell lines. Abbreviations used: ANOVA, analysis of variance; HSP, heat shock protein; HSPi, HSP inducer.

In sterlet sturgeon liver cells (Figure 4(a) and (d)), gills (Figure 4(b) and (d)), and kidneys (Figure 4(c) and (d)), HSP70 expression was evaluated both with and without inducing compounds under salinity stress conditions. The N800 + S13 group showed the greatest changes in HSP70 protein expression in liver, gill, and kidney cells. Compared to other inducing compounds, the protein expression was the lowest for the synthetic compound (SZ80). Compared with that under the control treatment, HSP70 protein expression under stress conditions (S13) slightly increased (*P* < 0.0001). In general, all three cell lines showed an increase in HSP70 expression when first treated with inducing compounds (especially N800) and then exposed to salt stress (*P* < 0.0001).

#### HSP90

**Fig. 5:**
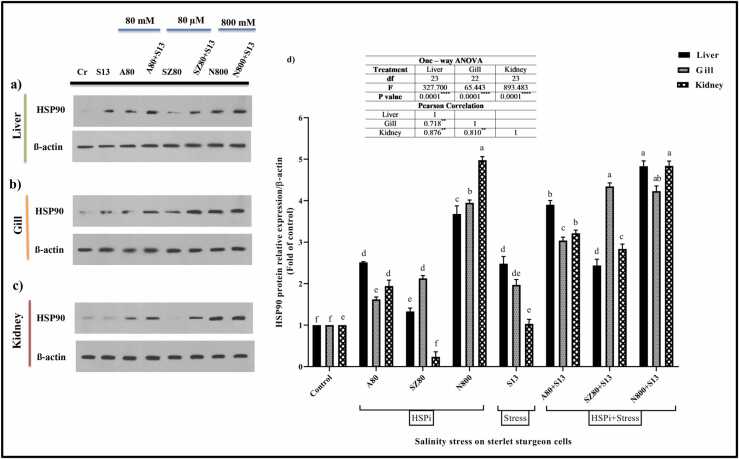
The relative expression of HSP90 using western blots. (a) Liver, (b) gill and (c) kidney cells. (d) Eight groups were considered: (1) control cells without treatment (Cr), HSPi groups containing (2) 80 mM amygdalin (A80), (3) 80 µM SZ (SZ80), (4) 800 mM Nopal endurance (N800), (5) 13 ‰ salinity stress group (medium salinity in the Caspian Sea; S13), and HSPi + salinity stress groups containing (HSPi + S13): (6) A80 + S13, (7) SZ80 + S13 and (8) N800 + S13. In eight treatment groups, each parameter was measured three times. HSP90 protein levels are shown as fold of control values (mean ± SEM), and within each column, different letters indicate significantly different groups using Duncan's test (a, b, c, and… *P* < 0.0001). Each of the three cell lines exhibited a positive correlation. Abbreviations used: ANOVA, analysis of variance; HSP, heat shock protein; HSPi, HSP inducer.

*Note*: Original images of the western blot gel are available in Supplementary Figures 4–7.

A change in HSP90 protein expression was observed in liver, gill, and kidney cells after the HSPi and salinity treatments (Figure 5; *P* < 0.0001). In the liver, the highest HSP90 expression was detected in the N800 + S13 treatment group (4.830-fold), and the lowest expression was detected in the control group. Compared with S13, N800 + S13 and A80 + S13 significantly enhanced HSP90 expression (Figure 5(a) and (d); *P* < 0.0001). In the gill, the SZ80 + S13 treatment groups (4.343-fold) and the control group had higher and lower HSP90 expression, respectively. Among all the HSPi compounds, A80 (1.620-fold) showed the lowest protein expression. Compared to the S13 group, the groups that received the inducing compounds first and then underwent salinity stress exhibited significantly increased HSP90 expression (*P* < 0.0001; Figure 5(b) and (d)). A similar pattern was observed in the kidney between the control group and the salinity group (S13). Among all HSPi compounds, N800 displayed the highest range of protein expression (4.976-fold). According to Figure 5(c) and (d), N800 + S13 (4.839-fold) significantly increased HSP90 expression compared to A80 + S13 (3.215-fold) and SZ80 + S13 (2.836-fold) (*P* < 0.0001).

### Biochemical parameters

The enzyme activities of liver cells exposed to salinity and inducers (HSPis) were different (Table 1; *P* < 0.0001). A similar pattern of changes was observed in aspartate aminotransferase (AST), alanine aminotransferase (ALT), alkaline phosphatase (ALP), and lactate dehydrogenase (LDH). A statistically significant increase in activity was observed for all enzymes mentioned in the S13 group compared to the control group (*P* < 0.0001). According to the results obtained for all HSPi compounds, N800 treatment resulted in the highest levels of enzymes (excluding ALT). After the inducers were applied, the groups were exposed to salinity (HSPi + S13), and the enzyme activity decreased significantly.

**Table 1:** Biochemical factors in the liver cells of sturgeon (mean ± SEM; *P* < 0.0001).

Groups*	Analysis of biochemical parameters (Unit per g protein total)			
	AST (U/g)	ALT (U/g)	ALP (U/g)	LDH (U/g)
Control	0.619 ± 0.042^e^	0.408 ± 0.010^e^	1.03 ± 0.011^d^	5.26 ± 0.206^d^
A80	0.709 ± 0.017^d^	0.463 ± 0.023^d^	0.996 ± 0.022^d^	5.79 ± 0.396^cd^
SZ80	0.7 ± 0.023^d^	0.530 ± 0.039^cd^	0.959 ± 0.018^d^	5.72 ± 0.255^cd^
N800	0.713 ± 0.026^d^	0.492 ± 0.010^d^	1.058 ± 0.010^d^	6.11 ± 0.278^c^
S13	1.295 ± 0.070^a^	1.042 ± 0.036^a^	2.193 ± 0.082^a^	10.26 ± 0.108^a^
A80 + S13	1.018 ± 0.041^b^	0.691 ± 0.030^c^	1.856 ± 0.052^b^	7.73 ± 0.286^b^
SZ80 + S13	0.838 ± 0.038^c^	0.583 ± 0.014^cd^	1.371 ± 0.039^c^	5.71 ± 0.328^cd^
N800 + S13	1.015 ± 0.031^b^	0.741 ± 0.036^b^	1.851 ± 0.044^b^	6.84 ± 0.242^bc^

Abbreviations used: ALP, alkaline phosphatase; ALT, alanine aminotransferase; AST, aspartate aminotransferase; HSPi, heat shock protein inducer; LDH, lactate dehydrogenase.

* Eight groups were considered: (1) control cells without treatment, HSPi groups containing (2) 80 mM amygdalin (A80), (3) 80 µM SZ (SZ80), (4) 800 mM Nopal endurance (N800), (5) 13 ‰ salinity stress group (medium salinity in the Caspian Sea; S13), and HSPi + salinity stress groups containing (HSPi + S13): (6) A80 + S13, (7) SZ80 + S13, and (8) N800 + S13. In each column, different letters on the numbers based on Duncan's test show a significant difference (a, b, c, and…).

Kidney cells had relatively similar AST, ALT, ALP, and LDH levels to liver cells (Table 2; *P* < 0.0001). AST and ALT showed patterns similar to those of ALP and LDH. A comparison of the S13 group with the control group revealed that the activity of these enzymes increased significantly (*P* < 0.0001). Among all the HSPi compounds (except ALP), SZ80 had the highest activity. The activity of AST, ALT, ALP, and LDH enzymes was lower in the S13 group than in the HSPi + S13 group (*P* < 0.0001).

**Table 2:** Biochemical factors in the kidney cells of sturgeon (mean ± SEM; *P* < 0.0001).

Groups*	Analysis of biochemical parameters (Unit per g protein total)			
	AST (U/g)	ALT (U/g)	ALP (U/g)	LDH (U/g)
Control	0.551 ± 0.007^f^	0.339 ± 0.023^d^	1.093 ± 0.012^e^	6.034 ± 0.179^e^
A80	0.605 ± 0.016^e^	0.317 ± 0.041^d^	1.015 ± 0.023^e^	5.70 ± 0.218^f^
SZ80	0.624 ± 0.023^e^	0.349 ± 0.036^d^	1.04 ± 0.020^e^	6.10 ± 0.105^bc^
N800	0.604 ± 0.011^e^	0.345 ± 0.012^d^	1.018 ± 0.012^e^	5.92 ± 0.172^e^
S13	1.199 ± 0.021^a^	0.828 ± 0.053^a^	2.25 ± 0.085^a^	10.29 ± 0.210^a^
A80 + S13	1.120 ± 0.025^b^	0.711 ± 0.022^b^	1.910 ± 0.053^b^	9.10 ± 0.268^d^
SZ80 + S13	0.881 ± 0.024^d^	0.621 ± 0.020^c^	1.50 ± 0.042^d^	7.30 ± 0.109^b^
N800 + S13	0.981 ± 0.044^c^	0.678 ± 0.023^bc^	1.743 ± 0.042^c^	7.87 ± 0.204^c^

Abbreviations used: ALP, alkaline phosphatase; ALT, alanine aminotransferase; AST, aspartate aminotransferase; HSPi, heat shock protein inducer; LDH, lactate dehydrogenase.

* Eight groups were considered: (1) control cells without treatment, HSPi groups containing (2) 80 mM amygdalin (A80), (3) 80 µM SZ (SZ80), (4) 800 mM Nopal endurance (N800), (5) 13 ‰ salinity stress group (medium salinity in the Caspian Sea; S13), and HSPi + salinity stress groups containing (HSPi + S13): (6) A80 + S13, (7) SZ80 + S13, and (8) N800 + S13. In each column, different letters on the numbers based on Duncan's test show a significant difference (a, b, c, and…).

### Cortisol levels

**Fig. 6:**
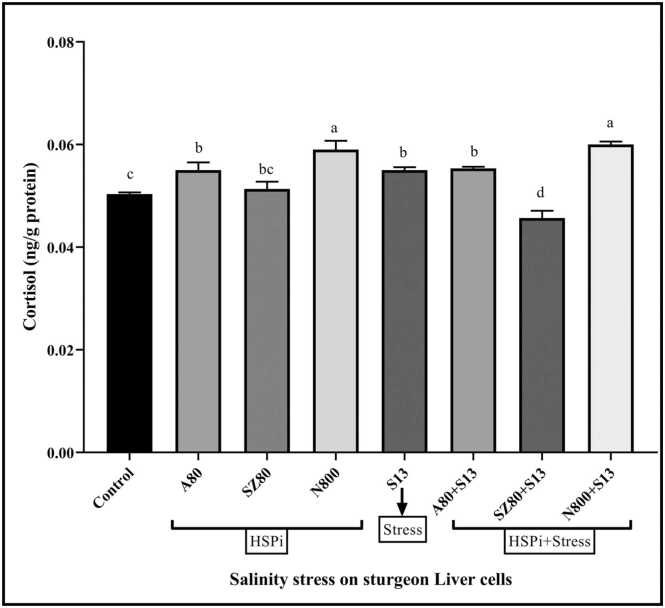
Assessing cortisol levels in *A. ruthenus* liver cells. Eight groups were considered: (1) control cells without treatment (Cr), HSPi groups containing (2) 80 mM amygdalin (A80), (3) 80 µM SZ (SZ80), (4) 800 mM Nopal endurance (N800), (5) 13 ‰ salinity stress group (medium salinity in the Caspian Sea; S13), and HSPi + salinity stress groups containing (HSPi + S13): (6) A80 + S13, (7) SZ80 + S13 and (8) N800 + S13. Generally, each parameter was measured three times in eight treatment groups. Compared with the control treatment, salinity treatment (S13) increased cortisol levels. However, treatment with N800 and N800 + S13 for 24 h increased cortisol levels. (Mean ± SEM) and within each column, different letters indicate significantly different groups using Duncan's test (a, b, c, and… *P* < 0.0001). Abbreviations used: HSP, heat shock protein; HSPi, HSP inducer.

Figure 6 showed changes in cortisol levels in the liver cells of sterlet sturgeons treated with HSPi and salinity (*P* < 0.0001). Compared to those in the control group, all groups showed an increase in cortisol levels in the presence and absence of inducing compounds (apart from SZ80 + S13; *P* < 0.0001). The N800 and N800 + S13 treatments exhibited the greatest changes. In the AMG groups, the change process was similar to that in the salinity group (S13; *P* < 0.0001).

### Antioxidant activity

As a result of measuring GST and GPx levels and TAC, the antioxidant activity of liver cells was assessed (Table 3; *P* < 0.0001). All treatment groups, except A80, demonstrated higher GST enzyme activity than did the control group. Groups S13 (0.259 U/g) and N800 + S13 (0.257 U/g) showed the highest specific activity of the GST enzyme (*P* < 0.0001). Among all the HSPi compounds, N800 (0.248 U/g) was associated with the highest GST activity (*P* *<* 0.0001). The N800 + S13 (2.354 U/g) group had more GPx enzyme activity, while the S13 (1.731 U/g) group had more GPx enzyme activity than did the control group (1.518 U/g) (*P* < 0.0001). The N800 (7.650 U/g) group showed a significant amount of TAC, and the S13 (5.450 U/g) group showed high TAC compared to the control group (5.346 U/g) (*P* < 0.0001). There was a significant amount of TAC in the N800 (7.650 U/g) group. Compared with the control (5.346 U/g), the S13 (5.450 U/g) group demonstrated high TAC activity (*P* < 0.0001). Overall, the enzyme activity in the A80 group was the lowest of all the inducing compounds (*P* < 0.0001).

**Table 3:** Antioxidant activity in liver cells of sturgeon (mean ± SEM; *P* < 0.0001).

Groups*	Analysis of antioxidant parameters (Unit per g protein total)		
	GST (U/g)	GPx (U/g)	TAC μmol/g
Control	0.204 ± 0.001^d^	1.518 ± 0.009^e^	5.346 ± 0.006^e^
A80	0.184 ± 0.002^e^	1.779 ± 0.006^d^	6.385 ± 0.006^d^
SZ80	0.217 ± 0.002^c^	2.125 ± 0.005^c^	6.685 ± 0.01^c^
N800	0.248 ± 0.002^ab^	2.203 ± 0.005^b^	7.650 ± 0.021^a^
S13	0.259 ± 0.001^a^	1.731 ± 0.004^d^	5.450 ± 0.012^de^
A80 + S13	0.225 ± 0.006^b^	2.229 ± 0.005^b^	6.450 ± 0.009^d^
SZ80 + S13	0.208 ± 0.001^d^	1.712 ± 0.008^d^	5.142 ± 0.008^f^
N800 + S13	0.257 ± 0.003^a^	2.354 ± 0.006^a^	7.273 ± 0.022^b^

Abbreviations used: GPx, glutathione peroxidase; GST, glutathione S-transferase; HSPi, heat shock protein inducer; LDH, lactate dehydrogenase; TAC, total antioxidant capacity.

* Eight groups were considered: (1) control cells without treatment, HSPi groups containing (2) 80 mM amygdalin (A80), (3) 80 µM SZ (SZ80), (4) 800 mM Nopal endurance (N800), (5) 13 ‰ salinity stress group (medium salinity in the Caspian Sea; S13), and HSPi + salinity stress groups containing (HSPi + S13): (6) A80 + S13, (7) SZ80 + S13, and (8) N800 + S13. In each column, different letters on the numbers based on Duncan's test show a significant difference (a, b, c, and…).

### Immune responses

We examined the activity of IgM, C3, and LYZ in liver cells exposed to different treatments, as shown in Table 4. Compared to the control (1.43 mg/g), the SZ80 + S13 (0.840 mg/g) treatment clearly decreased C3 activity. Compared to the control group, the S13 (1.14 mg/g) group had a lower C3 level, while the N800 + S13 (1.15 mg/g) group had a slightly greater C3 level (*P* < 0.0001).

**Table 4:** Immune responses in liver cells in sturgeon (mean ± SEM; *P* < 0.0001).

Groups*	Analysis of immune responses parameters (per g protein total)		
	C3 (mg/g)	IgM (mg/g)	Lysozyme (U/g/min)
Control	1.43 ± 0.031^a^	2.55 ± 0.026^a^	0.145 ± 0.002^a^
A80	1.19 ± 0.011^b^	1.84 ± 0.02^d^	0.107 ± 0.001^c^
SZ80	1.083 ± 0.014^c^	1.88 ± 0.005^d^	0.105 ± 0.0003^c^
N800	1.057 ± 0.008^c^	2.29 ± 0.02^b^	0.132 ± 0.0008^ab^
S13	1.14 ± 0.028^b^	2.03 ± 0.020^c^	0.121 ± 0.004^b^
A80 + S13	1.063 ± 0.017^c^	2.08 ± 0.02^c^	0.124 ± 0.004^b^
SZ80 + S13	0.840 ± 0.011^d^	1.5 ± 0.01^e^	0.084 ± 0.0008^d^
N800 + S13	1.15 ± 0.003^b^	2.60 ± 0.023^a^	0.145 ± 0.0008^a^

Abbreviations used: C3, component 3; HSPi, heat shock protein inducer; IgM, immunoglobulin M; LDH, lactate dehydrogenase.

* Eight groups were considered: (1) control cells without treatment, HSPi groups containing (2) 80 mM amygdalin (A80), (3) 80 µM SZ (SZ80), (4) 800 mM Nopal endurance (N800), (5) 13 ‰ salinity stress group (medium salinity in the Caspian Sea; S13), and HSPi + salinity stress groups containing (HSPi + S13): (6) A80 + S13, (7) SZ80 + S13, and (8) N800 + S13. In each column, different letters on the numbers based on Duncan's test show a significant difference (a, b, c, and…).

Compared to the control group (2.55 mg/g), the N800 + S13 group had higher levels of IgM (2.60 mg/g). Among the HSPi groups (A80: 1.84, SZ80: 1.88, and N800: 2.29 mg/g), none were more active than the S13 group (2.03; excluding N800). There was a significant difference between the N800 + S13, A80 + S13, and SZ80 + S13 treatments in terms of IgM levels (*P* < 0.0001). A similar change in LYZ enzyme activity was observed in the N800 + S13 group and the control group (0.145 U/g/min). Compared to that in the HSPi and HSPi + S13 groups, the overall enzyme activity decreased significantly (*P* < 0.0001).

### Principal component analysis

**Fig. 7:**
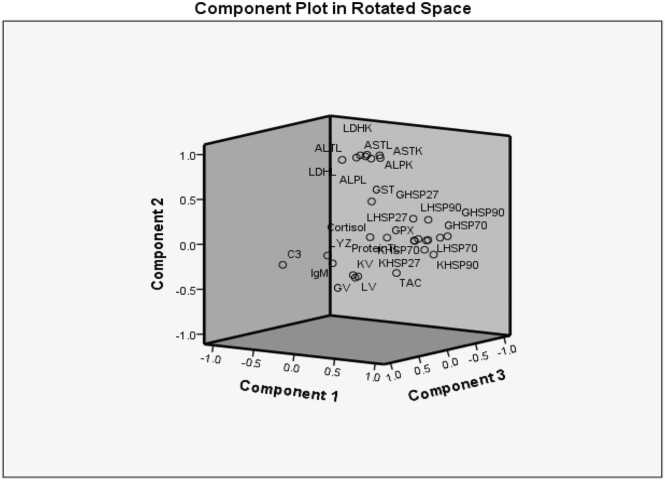
Principal component analysis (PCA) for salinity stress. Five components accounted for 95.20 % of the total variation. A significant relationship was found between the treatments and all variables measured under salinity stress for the first three components. Abbreviations used: G, gill; GPx, glutathione peroxidase; GST, glutathione S-transferase; GV, gill viability; IgM, immunoglobulin M; K, kidney; KV, kidney viability; L, liver; LV, liver viability; TAC, total antioxidant capacity.

Principal component analysis (PCA) was conducted on 29 variables and 24 treatments. The first 5 principal components accounted for 42.54 %, 25.25 %, 15.76 %, 7.94 %, and 3.70 % of the total variance, respectively (Table 5). Variables were used to interpret components with a score higher than 0.6 (Supplementary Data in Figure 8, Tables 1 and 2). For the first three components, all variables measured under salinity stress played a significant role in determining the relationships between the treatments and the tested variables (Figure 7). The enzyme variables liver-HSP90, liver-HSP27, GST, kidney-HSP70, kidney-HSP27, and (gill and liver) HSP70 were important factors in determining the relationship between treatments and the tested variables in the first component. In the second component, the main factor was TAC, and in the third component, the variables total kidney protein, total liver protein, kidney viability, and gill viability were significant. Finally, Pearson’s correlation analysis was performed between all measured parameters (Supplementary Table 3). The range of this correlation is between 0.988 and −0.071. The strongest significant positive correlation coefficients were observed between kidney ALP with liver ALP, kidney viability with liver viability, and kidney viability with gill viability, while the weakest significant negative correlation was reported between kidney viability with liver GST and gill viability with kidney LDH.

**Table 5:** Eigenvalue, percentage of variance and cumulative percentage of variance related to all five components.

	Component 1	Component 2	Component 3	Component 4	Component 5
Eigenvalue	12.37	7.32	4.57	2.30	1.07
Percentage of variance	42.54	25.25	15.76	7.94	3.70
Cumulative percentage of variance	42.54	67.79	83.55	91.49	95.20

## Discussion

In recent years, environmental stresses have increased, and traditional methods to deal with these stresses have not always been effective. Using non-stressful compounds to induce HSP is an innovative method that has recently gained attention and has been tested on several aquatic species.^9^, ^46^ The innovative application of HSPis to enhance cellular resilience represents a groundbreaking development in biotechnology. This novel approach leverages cells' intrinsic defense mechanisms, manipulating HSP expression to fortify cells against a myriad of stressors. Recent studies have illuminated the potential of HSPis like paracetamol to not only bolster the production of monoclonal antibodies but also to extend the viability and productivity of transgenic cell lines.^47^ Such advancements underscore the transformative power of HSP manipulation, heralding a new era in therapeutic and industrial bioprocesses.^48^

Both *in vivo* and *in vitro* studies combining different HSPis strains under salinity stress are very limited*. Acipenser ruthenus* was used as an ecological model to examine liver, kidney, and gill cell survival in the presence of HSPis and salinity. We hypothesized that inducing HSPs in isolated sterlet cells from the liver, gill, and kidney could increase protection and preparation against salinity stress. First, using an MTT assay, it was found that salinity negatively affects sterlet cell viability (Figure 2). Moreover, salinity treatment altered HSP27, HSP70, and HSP90 expression in all three cell lines (Fig. 3, Fig. 4, Fig. 5, respectively). Salinity increased the levels of biochemical enzymes, including AST, ALT, ALP, and LDH, in liver and kidney cell lines (Tables 1 and 2, respectively). Additionally, it enhanced cortisol levels in liver cells (Figure 6). Finally, salinity stress increased GST, TAC, and GPx enzyme oxidative activity (Table 3). The immune parameters IgM, LYZ, and C3 were also reduced (Table 4).

Despite our initial findings about salinity stress, we encountered significant challenges. (1) HSPs can cause the same alterations as salinity without reducing cell viability or increasing cell death. (2) The combination of HSP and salinity can restore normal conditions. (3) HSP expression is associated with cell viability in *A. ruthenus.*

An experiment was conducted to determine the effects of salinity stress on the early life stages of zebrafish (*Danio rerio*). A salinity gradient was applied to zebrafish embryos at different developmental stages to evaluate the objectives. Results showed that zebrafish embryos (2–4-cell stage) survived hatching at 2 ppt (hatching rate 54.5 %), but died at 4 ppt (hatching rate 23.5 %). Embryos exposed to different salinities (e.g., 0, 6, 8, 10, and 12 ppt) and then returned to freshwater (0 ppt) could hatch up to 8 ppt only after 60 min, but not after 120 min.^49^ Researchers have found that increasing HSP levels *in vivo* through non-lethal heat shock, exogenous HSPs, or herbal compounds could create protective immunity and increase survival in crustaceans.^50^ According to several studies, survival and HSP expression are directly related to stress conditions.^41^, ^51^, ^52^ As HSP chaperones play a crucial role in protecting cells against stress, we investigated whether HSPis could counteract the effects of salinity on *A. ruthenus* cells. To test this hypothesis, we used NOP, AMG, and SZ (a pirano-piranazole-derived compound, Supplementary Figure 1). To better understand this challenge, we examined the viability of cells after the mentioned compounds were applied. MTT assays (Supplementary Figure 2) revealed that NOP decreased cell viability, while AMG and SZ increased cell viability. The viability of cells first treated with inducing compounds and then exposed to salinity also increased (HSPi + S13; Figure 2).

Fish farming focuses on the first challenge. One way to conserve endangered fish populations is to raise them on special farms and release them into freshwater after they mature.^53^ When fish are raised under optimal conditions on farms, the water conditions they are exposed to after release will vary, which is a disadvantage of this approach.^54^ Therefore, using HSPis can alleviate this problem by addressing the first aspect. However, the second aspect of this challenge, which involves combining salinity with HSPis, is crucial for reducing or eliminating salinity stress.^9^, ^55^ A study was conducted on the valuable species *Ruditapes philippinarum* (Rp) under stress. In thermal and salinity stress conditions, RpHSP70 expression in gills was induced, and the increase in RpHSP70 Messenger Ribonucleic acid likely reflected the need for more HSP70 proteins to refold and renaturate abnormal proteins. These findings will help better understand the biological function of HSPs in defending against environmental challenges and the innate immune response in *R. philippinarum*.^56^ In this study, we observed a notable increase in all HSPs (HSP27, HSP70, and HSP90) expression in cells treated with NOP and AMG compared with the control and salinity (S13) groups. In kidney cells, the SZ synthetic compound reduced HSP expression but performed better when combined with salinity stress (Fig. 3, Fig. 4, Fig. 5, respectively). Our results indicate that NOP and AMG can be utilized as HSPis in fish cells. In addition, when comparing S13 and HSPi + S13 treatments, we found that the cells that first received the HSPis and then were subjected to salinity stress (HSPi + S13) had higher HSPs protein expression levels. Upregulation of HSP is an important coping mechanism for fish to deal with stress and to protect their cells from damage. By more expressing HSP, organisms can mitigate climate change effects, including prolonged periods of high ambient temperature and salinity.^57^

Fish stress levels can be indicated by the activity of biochemical enzymes (AST, ALT, ALP, and LDH).^58^ A study was conducted on *Cyprinus carpio* under salinity stress. After exposure to salinity stress, AST, ALT, ALP, and LDH enzyme activity increased, which indicates a need to deal with stress and cell damage.^59^ Furthermore, in another study performed on pufferfish (*Takifugu obscurus*) under stress, AST, ALT, LDH, glucose, and triglyceride levels increased significantly while ALP levels decreased. Additionally, it reduced the number of blood cells, inhibited cell viability, and caused DNA damage and apoptosis.^60^ According to the present study, salinity (S13) can lead to an increase in biochemical parameters, leading to cell stress and death. When liver and kidney cells were pretreated with inducing compounds, especially SZ (SZ80 + S13), there was a significant decrease in liver and kidney enzymes (Table 1, Table 2, respectively).

Previous studies have demonstrated a clear correlation between cortisol activation and HSP expression under stressful conditions.^32^, ^33^, ^61^ During fish migration from freshwater to saltwater, cortisol plays a critical role in regulating osmosis.^62^ Current studies have shown that cortisol changes in liver cells treated with AMG alone or in combination with salinity (A80 + S13) are similar to those in liver cells treated with salinity alone (S13). In addition, cortisol levels increased in the NOP treatment group and decreased in the SZ treatment group (Figure 6).

Induced Reactive oxygen species production has been linked to salinity changes, which can adversely affect immune function and fish health.^63^ Salinity also affects the activity of antioxidant enzymes, particularly GST, GPx, and TAC.^64^ Previous studies have shown a clear relationship between antioxidant parameters, especially GST activity, and HSP expression.^65^, ^66^ In the present study, we found that the presence of HSPi compounds, especially Nopal and AMG, in combination with salinity (HSPi + S13) increased antioxidant enzyme activity (Table 3). Our results suggested that the expression of the studied HSP proteins and antioxidant activity were directly related. Salmonids, tilapia, and sea bream exhibit species-specific immune responses to salinity changes.^67^ Changing salinity activates the innate immune system of pipefish as a general stress response.^62^ Another study conducted on sturgeon fish showed that immune activity in fish was moderated in the presence of HSPi.^9^ In our study, we found that optimal results for the immune response (C3, IgM, and LYZ) were obtained in cells first treated with NOP and then subjected to salinity stress (N800 + S13; Table 4).

HSP expression in different tissues was related to the most studied parameters according to PCA and correlation analysis (Table 5, Supplementary Tables 1–3). This highlights the importance of this family in different biological states. Using PCA, we identified the most effective treatment for salinity stress. Based on this analysis, we found that HSP expression was directly related to antioxidant parameters (GPx and TAC), immune response, and biochemical enzymes (Figure 7). Therefore, the combination of NOP (N800 + S13) and AMG (A80 + S13), especially NOP, can control salinity effects and return the parameters to normal.

## Conclusion

Our study investigated how HSPis could modulate salinity-induced cell death in *A. ruthenus* sturgeon cells. Based on our findings, HSPis can positively affect HSP expression, immune responses, and antioxidant activity. Furthermore, they can increase the activity of cortisol and biochemical enzymes in *A. ruthenus* in response to salinity, thereby enhancing cell survival *in vitro*. The use of HSPis may be a powerful and reliable way to increase salinity stress in *A. ruthenus* and reverse its harmful effects. To effectively apply this approach to real-life situations, particularly *in vivo* and in combination with other stresses, and to understand the effects of these ecological factors on the health status of the species, further research is needed.

## Materials and methods

### The synthesis of a new compound (SZ)

In the Supplementary Data, a detailed explanation of the synthesis of 4,4-(4,1-phenylene)bis(5-amino-3-methyl-4,1-dihydropyrano[3,2-*c*]pyrazole-6-carbonitrile was provided, as shown in Supplementary Figure 1(a)–(c).^41^, ^68^

### The isolation of cells from liver, gill, and kidney tissue

The International Sturgeon Research Institute provided a specimen of *A. ruthenus* measuring 18 cm in length and weighing 15.28 g. According to Zarei *et al.*,^46^, ^52^ liver, gill, and kidney tissue cells were extracted and analyzed.

### Determination of the optimal HSPi dose

#### Treatment with HSPis and 3-(4,5-dimethylthiazol-2-yl)-2,5-diphenyl-2H-tetrazolium bromide assay

This section is fully described in the Supplementary Data (Section 1.2). Based on the results of the 3-(4,5-dimethylthiazol-2-yl)-2,5-diphenyl-2H-tetrazolium bromide (MTT) assay, the following optimal doses of HSPi were selected: 800 mM NOP (Source Naturals, Inc, Santa Cruz, CA; N800), 80 mM AMG (Sigma–Aldrich; A80), and 80 μM SZ (SZ80) (Supplementary Figure 2(a)–(c), respectively).^41^, ^69^

#### Salinity treatment groups

According to previous studies, the Caspian Sea has an average salinity of 13 parts per trillion (ppt).^7^, ^9^ The same concentration was used in this study, and the salt-containing medium was autoclaved (S13). The 5 × 10^5^ cells/mL were counted and transferred to a 24-well plate with culture medium (90 % Dulbecco's Modified Eagle Medium, 10 % Fetal bovine serum, 100 U/mL streptomycin/penicillin (1 %) and 100 U/mL amphotericin B (1 %)). The plates were incubated at 22 °C, 5 % CO_2_, and 95 % humidity. After 24 h, the culture medium was replaced (with 1 % FBS), and the cells were divided into 8 groups (Figure 1): (1) control (without any S13 or HSPi); (2) cells that separately received HSPi (2-1-N800, 2-2-A80, and 2-3-SZ80); (3) cells subjected to only S13; and (4) cells that separately received HSPi and then underwent S13 treatment: 4-1-N800 + S13, 4-2-A80 + S13, and 4-3-SZ80 + S13. Each group was repeated three times. Finally, after 24 h, the cells were centrifuged (at 4 °C, 5000 rpm for 5 min). The final volume was 500 µL. In all cases, aseptic conditions were used.

The protein concentration was determined using the Bradford assay,^70^ with bovine plasma albumin (Sigma–Aldrich) serving as the standard (Supplementary Figure 3).

### Western blotting analysis

In liver, gill, and kidney cells, HSP27, HSP70, and HSP90 expression patterns were studied using the western blotting technique described by Werner *et al.*^71^ Briefly, sodium dodecyl sulfate–polyacrylamide gel electrophoresis was performed on equal amounts of protein (25 μg) from each sample (12 % polyacrylamide gel and 5 % stacking gel). The gels were then electroblotted onto a total polyvinylidene fluoride transfer membrane (Millipore, Bedford, MA). Primary antibodies against HSP27, HSP70 and HSP90 were used for immunoblotting (sc-13132 (Santa Cruz Biotechnology, Inc.), H5147, and H1775 from Sigma–Aldrich). For ß-actin, which is a housekeeping protein (control), monoclonal anti-ß-actin (A3854) and anti-mouse IgG–peroxidase conjugate (A2304, Sigma–Aldrich) were utilized as primary and secondary antibodies, respectively. 3,3′-Diaminobenzidine (Sigma–Aldrich, D-7304) and H_2_O_2_ were used as substrates.

*Note*: Gill cells were analyzed using western blots, and the enzyme assay was not performed on these cells.

### Measurement of biochemical enzymes

According to the protocol provided with the kit (MyBioSource, USA), liver and kidney cells were examined for AST, ALT, ALP, and LDH activity.^72^, ^73^

### The detection of cortisol

Cortisol level was measured in liver cells using an enzyme-linked immunosorbent assay (ELISA) kit (Nanjing Jiancheng Institute, Nanjing, China) in accordance with the manufacturer's instructions. A spectrophotometric measurement (450 nm) was used to detect the color changes.^73^, ^74^

### Antioxidant assay

The GST activity was determined by increasing the absorbance at 340 nm with 1 mM 1-chloro-2,4-dinitrobenzene (Sigma–Aldrich) and 1 mM GSH in 100 mM Na-phosphate buffer, pH 6.5.^75^

Following the oxidation of Nicotinamide adenine dinucleotide phosphate in 100 mM Na-phosphate buffer (pH 7.5), 1 mM Ethylenediaminetetraacetic acid, 0.12 mM NADPH, 2 mM GSH, 1 mM NaN3, 1 U glutathione reductase and 0.6 mM H_2_O_2_, GPx activity was measured at 340 nm (ZellBio GmbH, Germany).

Similar to GPx, TAC levels were assessed using an ELISA kit (ZellBio GmbH, Germany) at 490 nm.^76^

### Immune responses

IgM levels were measured using an ELISA quantification kit (Hangzhou, Zhejiang, Eastbiopharm Co., Ltd., China).^77^ IgM standards and supernatant samples were analyzed manually. After all the necessary steps, the Optical density at 450 nm was determined within 15 min. The measurements were repeated three times.

C3 levels were determined using an ELISA sandwich with a fish ELISA kit (Hangzhou, Zhejiang, Eastbiopharm Co., Ltd.).^30^ The supernatant consisted of an antibody-enzyme monoclonal well previously coated with a C3 fish monoclonal antibody. The plate was incubated at 37 °C, after which the biotin-labeled C3 antibody was mixed with streptavidin–horseradish peroxidase to form an immune complex. The plate was washed to remove the unmixed enzyme. The liquid color changed to yellow upon the addition of sulfuric acid. The OD at 450 nm was measured using a microplate reader, and the C3 concentrations were expressed in ng/mL.

Using a turbidimetric method, LYZ activity was assessed.^78^ *Micrococcus lysodeikticus* (Sigma–Aldrich) was used as a substrate (0.2 mg/mL 0.05 M phosphate buffer, pH 6.6). A standard curve was constructed using lyophilized chicken egg white LYZ, and the rate of change in turbidity was measured at 530 nm. The results corresponded to the LYZ activity of chicken egg whites.

### Statistical analysis

Statistical analysis was performed using SPSS/PC + 23 (SPSS Inc.) and GraphPad Prism 8. All numerical data are presented as the mean ± standard error of the mean. First, the normality of the data for each group was checked and confirmed using the one-sample Kolmogorov–Smirnov test. To analyze significant differences between groups, one-way analysis of variance and Duncan's test for multiple comparisons were used. PCA was performed to evaluate the differences in the main components between the studied parameters and different treatments for salinity stress. A correlation analysis was conducted to examine the possibility of a relationship between the biomarkers and fish cells. The significance level was set at *P* < 0.0001.

## Consent for publication

All of the authors have read and approved the paper for publication.

## Author contributions

*Sevda Zarei*: Performed the experiments, wrote the original draft, and performed the statistical analysis. *Hossein Ghafouri*: Conceptualization, supervision, methodology, writing – review & editing. *Leila Vahadatiraad*: Performing the experiments. *Behrooz Heidari*: Methodology, Advisor, writing – review & editing.

## Ethics statement

The authors declare that they have no known competing financial interests or personal relationships that seem to affect the work reported in this article. We declare that we have no human participants, human data, or human tissues.

## Funding and support

This work was supported by the Iranian National Science Foundation (INSF, grant no. 4028365).

## Declarations of interest

The authors declare the following financial interests/personal relationships which may be considered as potential competing interests: Sevda Zarei reports financial support was provided by Iran National Science Foundation. If there are other authors, they declare that they have no known competing financial interests or personal relationships that could have appeared to influence the work reported in this paper.

## Data Availability

The data are available upon request.
