# Supplementary material for: The influence of HSP inducers on salinity stress in sterlet sturgeon (Acipenser ruthenus): In vitro study on HSP expression, immune responses, and antioxidant capacity
Source: Cell Stress Chaperones. 2024 Jun 22;29(4):552–66. doi: 10.1016/j.cstres.2024.06.004 (PMC11268179; doi:10.1016/j.cstres.2024.06.004)
Supplement: Supplementary file 1 — Supplementary material [file mmc1.docx]

***1. Supplementary data***

***1.1. Synthesis of compound SZ***

The compound 4,4-(4,1-phenylene) bis (5-amino-3-methyl-4,1-dihydropyrano[3,2-c] pyrazole-6-carbonitrile (SZ) was synthesized by reacting 1 mmol of pyrazolone with 1.1 mmol malonitrile and 1 mmol terephthaldehyde in the presence of 5% NaOH as catalyst in ethanol solvent under reflux conditions. A chilled distilled water bath was placed over the balloon to complete the reaction. Using filter paper the precipitate was dried in an oven. The chemical composition was recrystallized in ethanol for further purification. UV-Visible, FT-IR and NMR spectroscopic analysis (Supplementary Fig. 1a, 1b and 1c, respectively) confirmed the structure of the synthesized compound. Its molecular weight 427 gr/mol is assigned.

***1.2. Treatment by HSP inducers and MTT assay***

5×10^5^ cells/ml from each tissue (liver, kidney, and gill) were counted and transferred to a plate with 96 wells. The cells were incubated at 22°C, 5% CO_2_, and 95% humidity in Dulbecco's Modified Eagle Medium (DMEM) containing 10% FBS, 100 U/ml streptomycin/penicillin (1%) and 100 U/ml Amphotericin B (1%). After 24 hours, the medium was replaced with 1% FBS containing DMEM. Then, HSPi was as follows: Tex-OE^®^ (NOP): 0, 50, 100, 200, 400, 800 and 1600 mM, amygdalin (AMG; Sigma- Aldrich): 0, 1.25, 2.5, 5, 10, 20, 40 and 80 mM and also SZ: 0, 5, 10, 20, 40, 80 and 160 µM for 24 h. A group without treatment was considered a control group. Afterward, the cells' viability was assessed via MTT assay. The final volume was 200 µl.

By reducing the brightly colored tetrazolium salt to its formazan form, a purple-blue color is produced, whose intensity can be measured spectrophotometrically. In practice, at the end of the treatment period, the medium was replaced with 10% of 3-(4,5-dimethylthiazol-2-yl)-2,5-diphenyl-2H-tetrazolium bromide solution (MTT reagent: 5 mg/ml, Sigma-Aldrich) and the plate was incubated at 22° C, 5% CO_2_ and 95% humidity for 2.40- 4 h. The supernatant was removed and the formazan crystals formed were solubilized in 100 μl of dimethyl sulfoxide (DMSO; Sigma-Aldrich) for 30 min. The absorbance at 570 nm was measured and the results were expressed as a percentage of the control (which is considered 100%). Based on the results of the MTT assay the optimal doses of HSPi were selected: 800 mM NOP (N800), 80 mM AMG (A80) and 80 μM SZ (SZ80) (Supplementary Fig. 2a, 2b and 2c, respectively). The viability was calculated using the following formula:

$$Cell viability = [ \frac{\mathrm{OD} (treatment)}{\mathrm{OD} (control)}]\times100\%$$

***1.3. Total protein***

Total protein changes in liver and kidney cells treated with inducing compounds and salinity stress were investigated (Supplementary Fig. 3). The change process was similar. In both cell lines, the highest value was observed in the SZ80+S13 group (P˂0.0001).

***1.4. Original photos of the western blot gel***

Pictures related to the expression of HSP27, HSP70 and HSP90 proteins in liver, gill and kidney cells (Section 3-2).

***1.5. Principal Components Analysis (PCA)***

One of the statistical methods for analyzing data is factor analysis. Additional data related to principal component analysis (PCA) in salinity stress are reported in Tables 1 and 2 (Section 3-7). The scree-graph diagram (Supplementary Fig. 8) shows the relationship between the eigenvalues and the factors. From the fifth factor onwards, the eigenvalues are very reduced so it can be concluded that the first five factors play the most significant role in explaining the variance of the samples. Also, a Pearson correlation analysis was performed between all the parameters measured (Table 3).

**Figure legends**

**Figure 1. FT-IR, H-NMR, and C-NMR spectra for SZ**. **a)** FT-IR (KBr, Cm-1) ν max: 3390 (NH); 3302 (NH2); 3168 (ArCH); 2923-2956 (Alkyl CH); 2186 (CN); 1645, 1597 (Ar C=C). **b)** H-NMR (400 MHz, DMSO-d6): δ (PPM); 1.75 (S, 6H, CH3), 4.58 (s, 2H, CH), 6.87 (s, 4H, NH2), 7.12 (s, 4H, ArH), 12.1 (s, NH). **c)** 13C NMR (100 MHz, DMSO-d6): d (ppm) 10.18, 36.42, 57.67, 98.18, 121.28, 128.12, 136.07, 143.24, 155.18, 129.01, 161.30.

**Figure 2. Viability (%) of cells isolated from *Acipenser ruthenus* tissues (Mean ± SEM) and determining the optimal dose for HSPi.** Liver, gill and kidney of cells were treated with **a)** (0-1600) mM of Nopal Endurance (NOP), **b)** (0-80) mM of Amygdalin (AMG), **c)** (0-80) µM of SZ.

**Figure 3. Analyzing the total amount of protein in *A. ruthenus* liver and kidney cells.** 8 groups were considered: 1) control cells without treatment (Cr), HSPi groups containing: 2) 80 mM Amygdalin (A80), 3) 80 µM SZ (SZ80), 4) 800 mM Nopal Endurance (N800), 5) 13‰ of salinity stress group (the medium salinity in the Caspian Sea; S13), HSPi+ salinity stress groups containing (HSPi+S13): 6) A80+ S13, 7) SZ80+ S13 and, 8) N800+ S13. In eight treatment groups, each parameter was measured three times. The vertical bar denotes a standard error (Mean± SEM) and within each column in the chart, different letters indicate significantly other groups using Duncan's test (a, b, c, and...; P < 0.0001). There was a positive correlation between changes in total protein in liver and kidney cells.

**Figure 4. HSP27. a)** Liver**, b)** Gill and **c)** Kidney cells.

**Figure 5. HSP70. a)** Liver**, b)** Gill and **c)** Kidney cells.

**Figure 6. HSP90. a)** Liver**, b)** Gill and **c)** Kidney cells.

**Figure 7. ß-Actin. a)** Liver**, b)** Gill and **c)** Kidney cells.

**Figure 8. Scree Plot.**

**Figures**

**Fig. 1**

**a)**


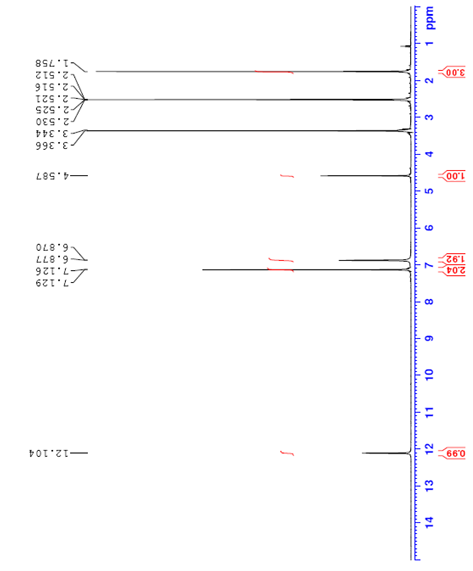
**
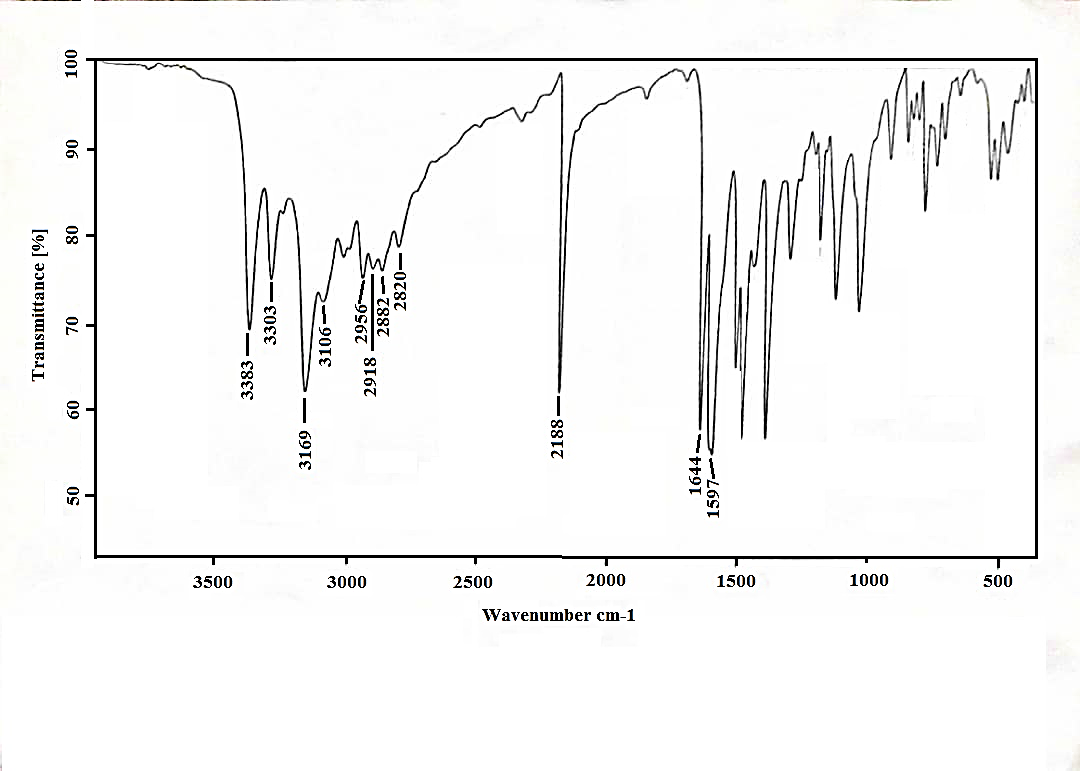
**

**b)**


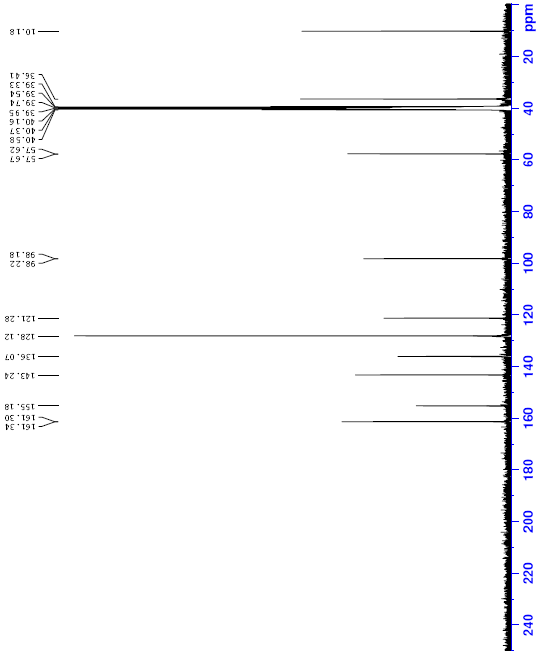
**c)**

**2.**

**2. a)**

**
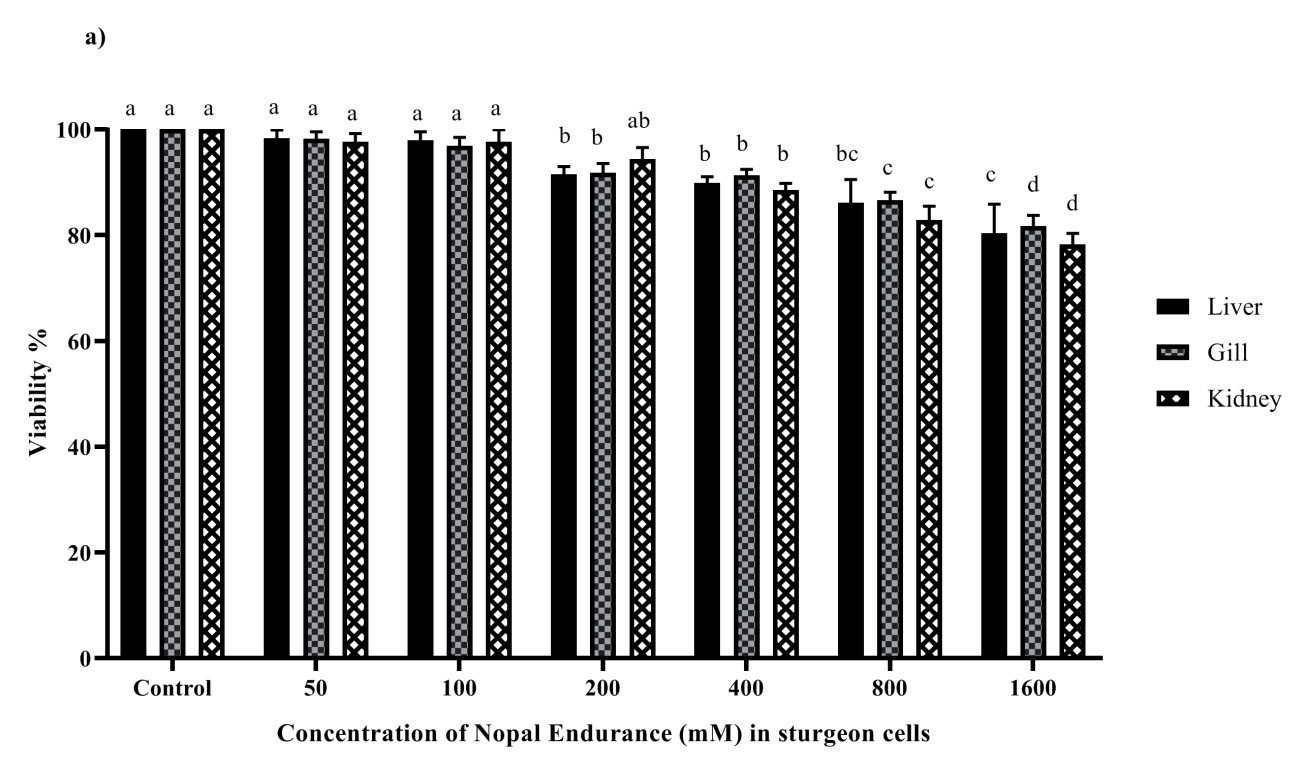
**

**2. b)**

**
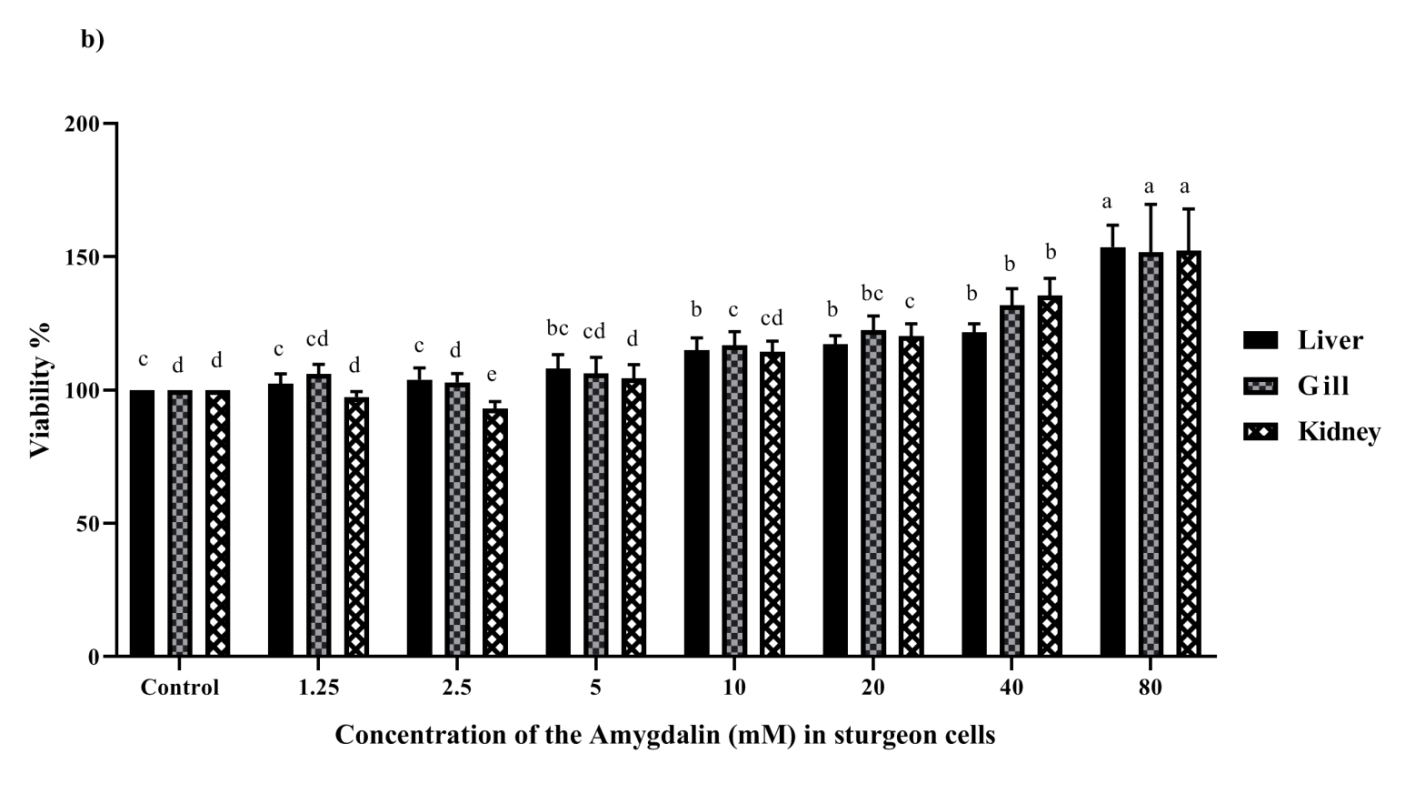
2. c)**

**
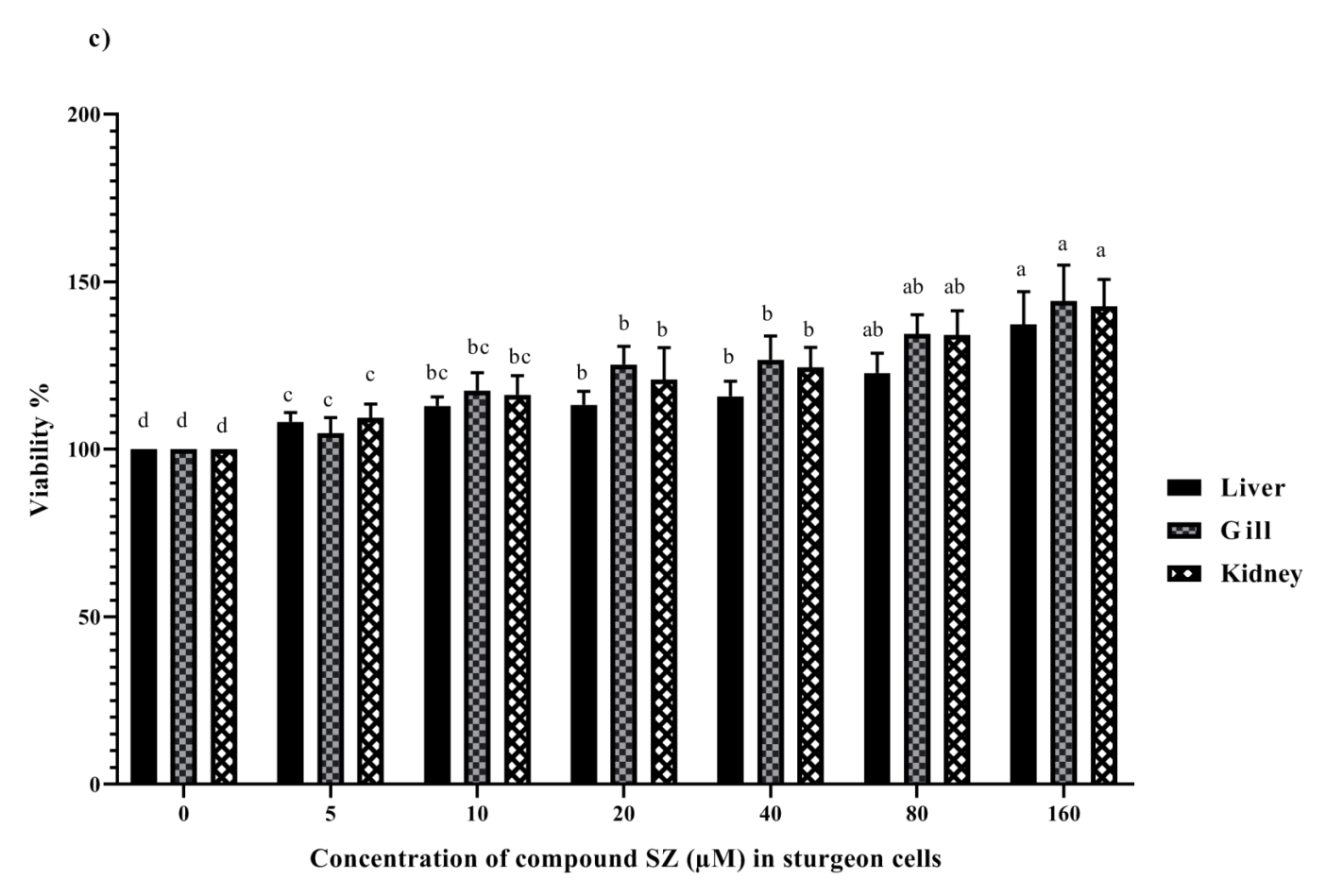
**

**Fig. 3**

**
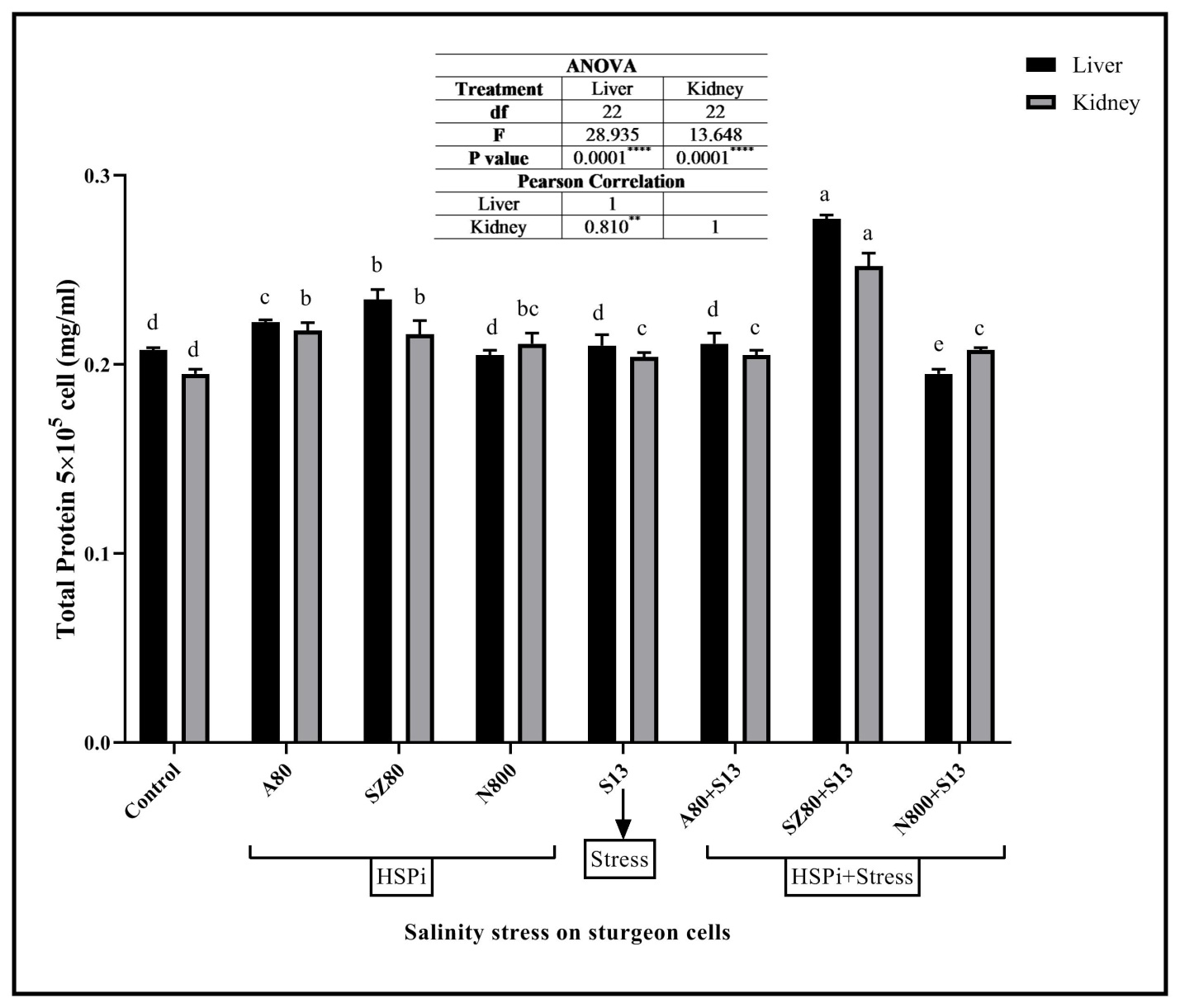
**

**Fig. 4**

**a)**

**
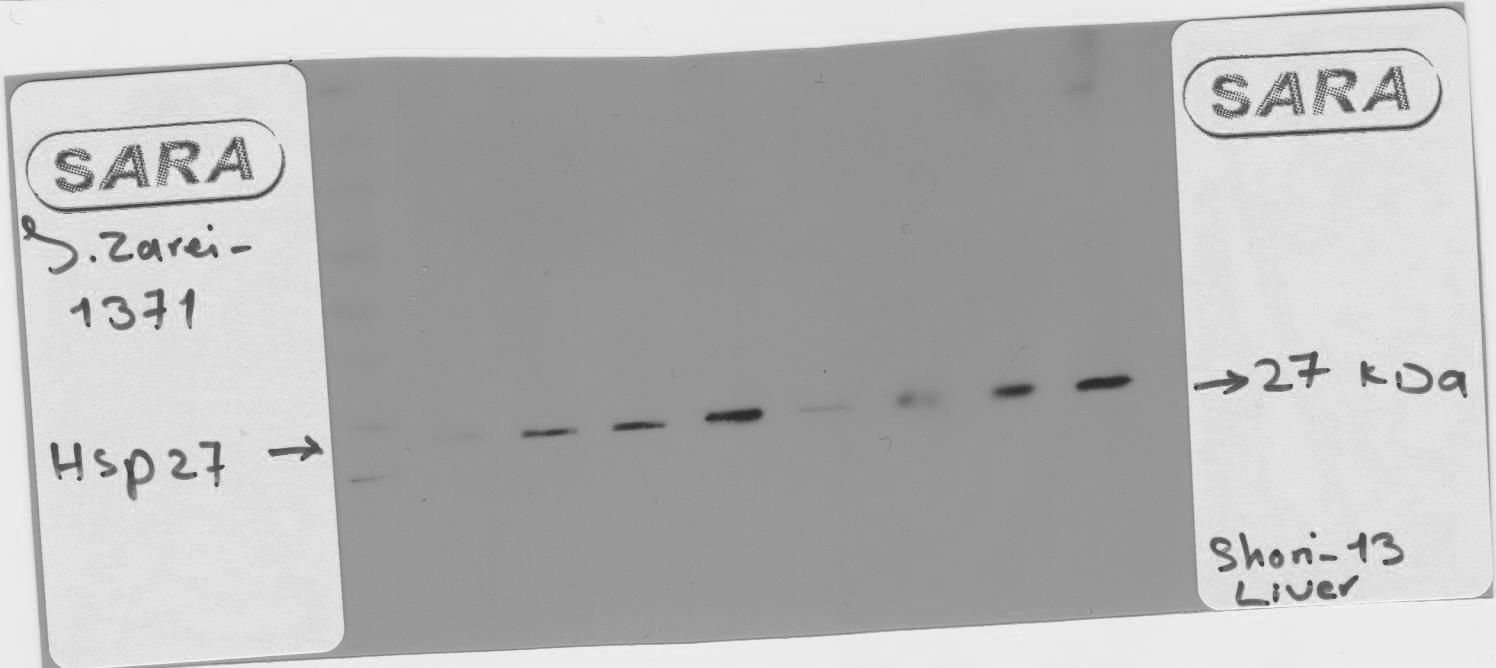
**

**b)**

**
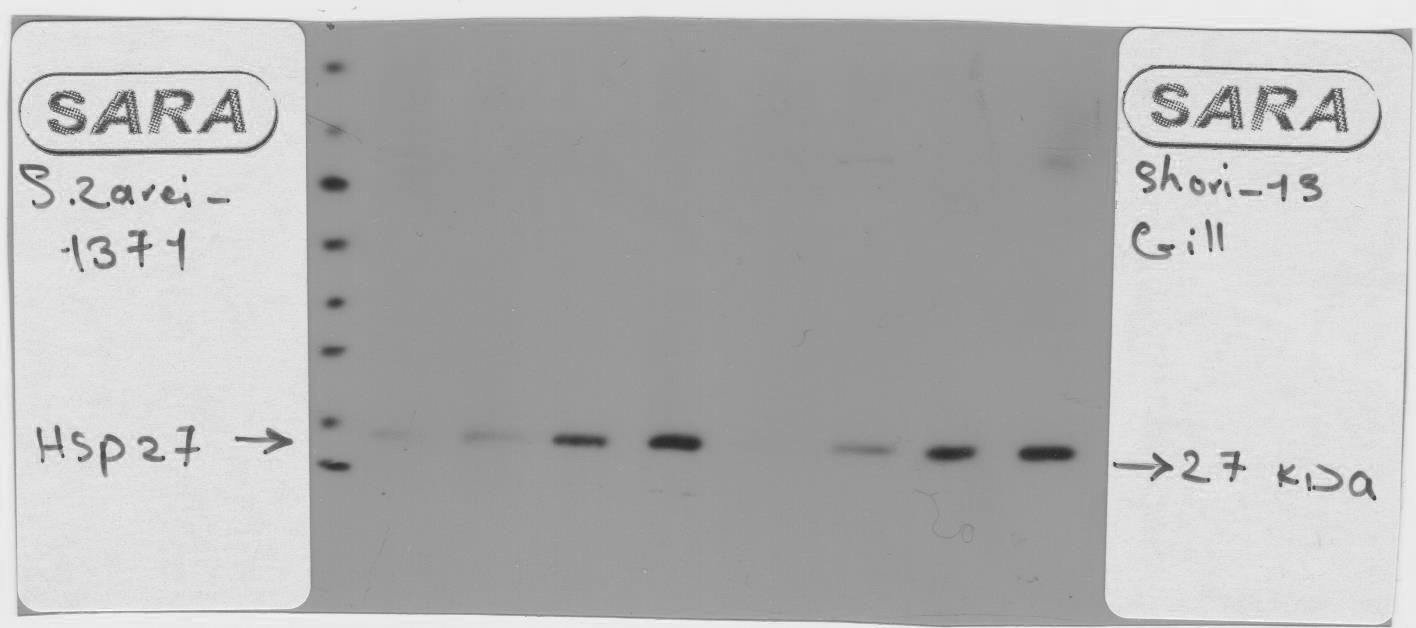
**

**c)**

**
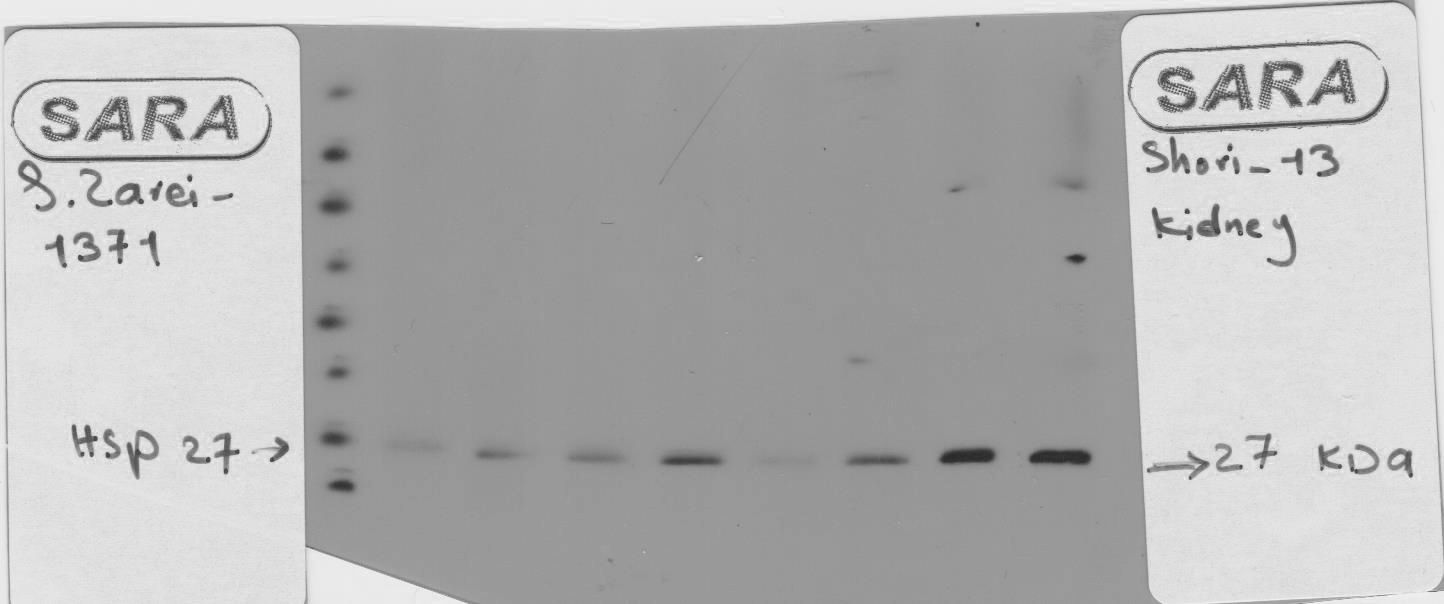
**

**Fig. 5**

**a)**

**
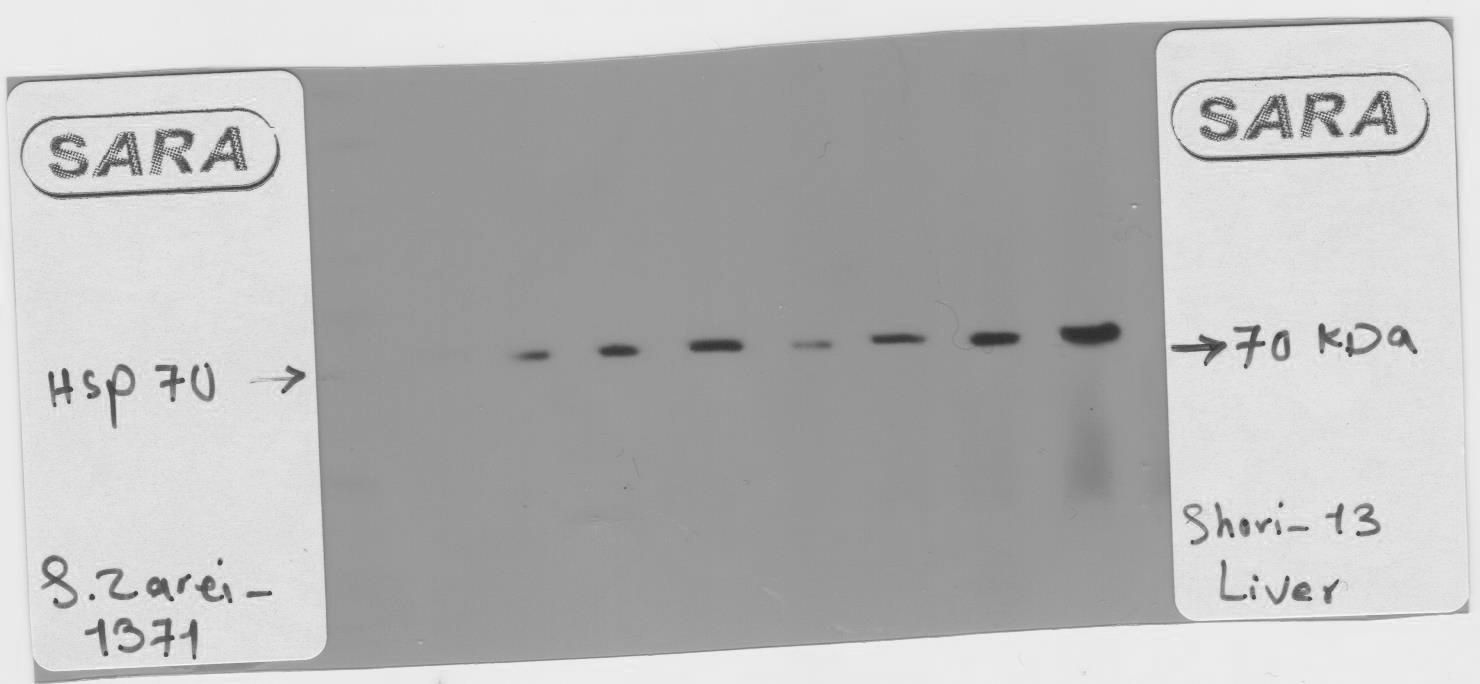
**

**b)**

**
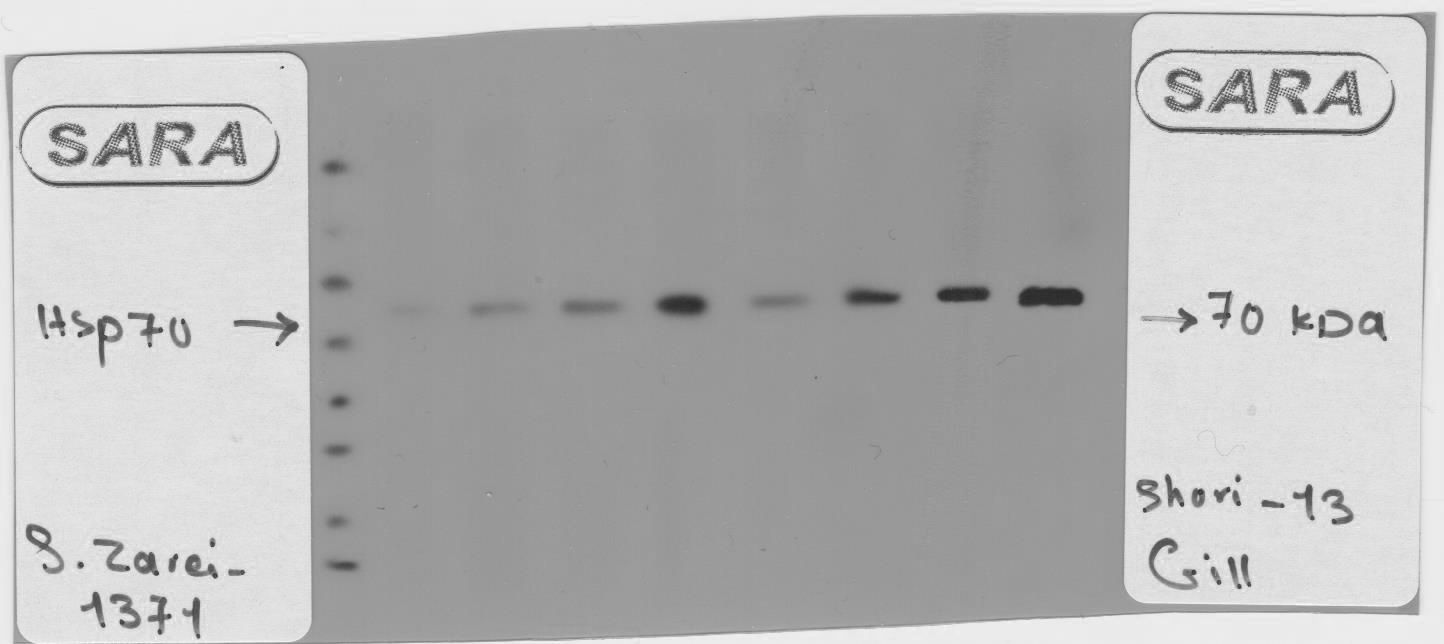
**

**c)**

**
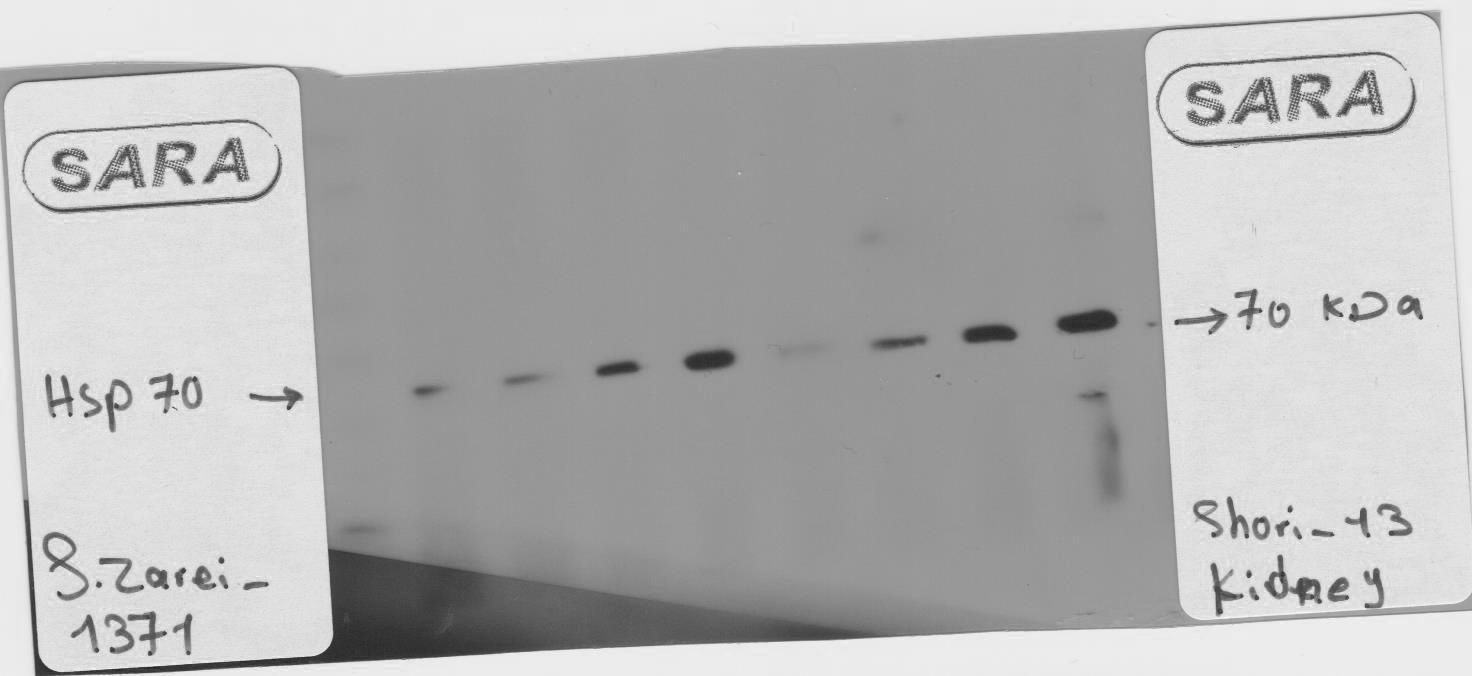
**

**Fig. 6**

**a)**

**
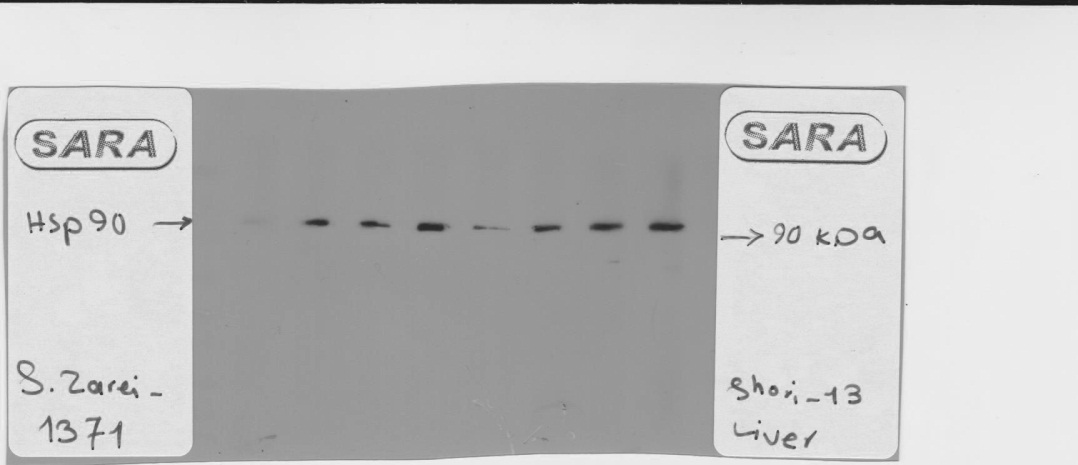
**

**b)**

**
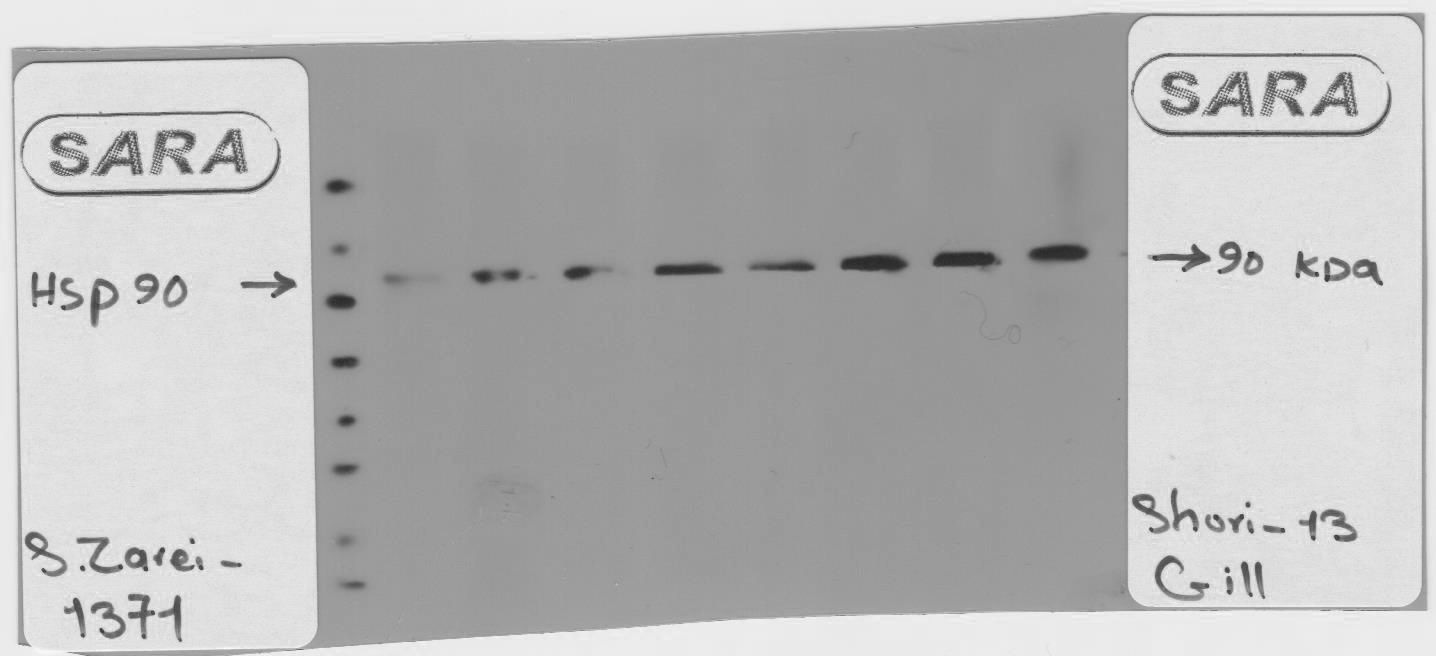
**

**c)**

**
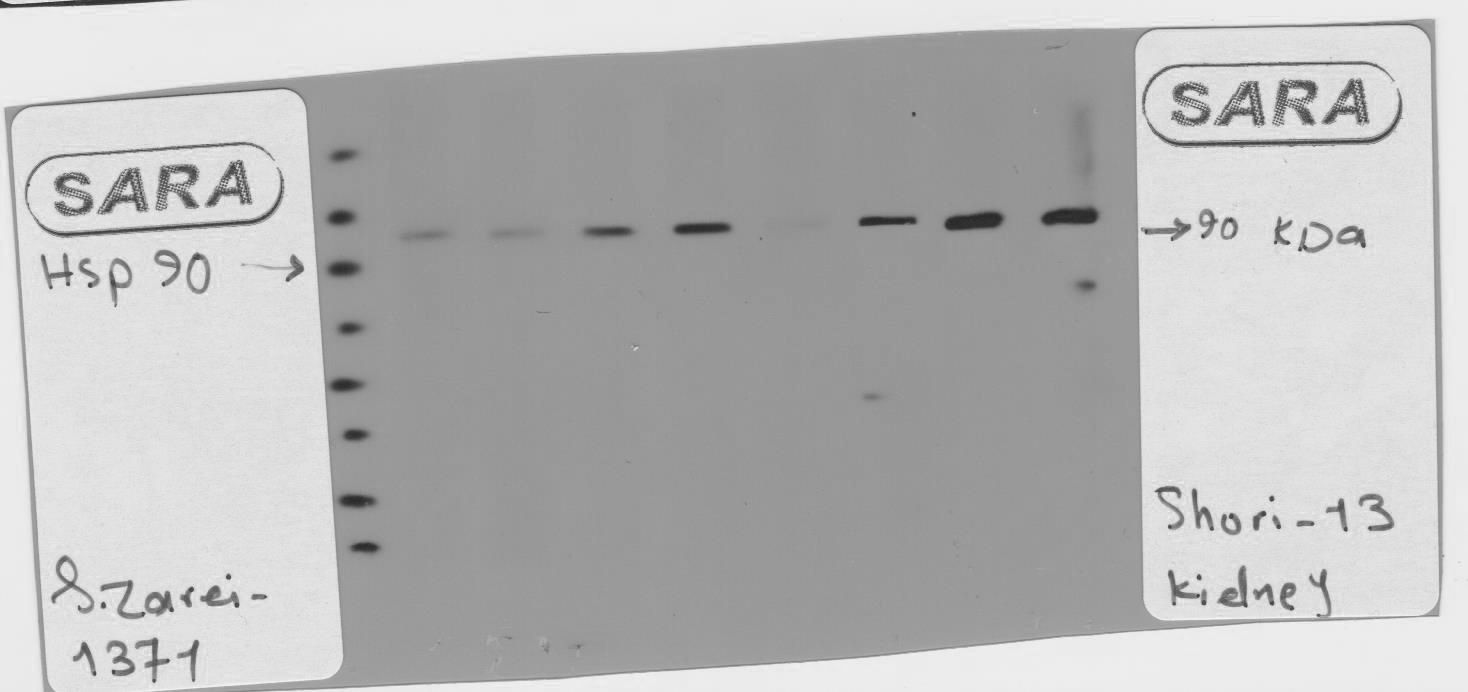
**

**Fig. 7**

**a)**

**
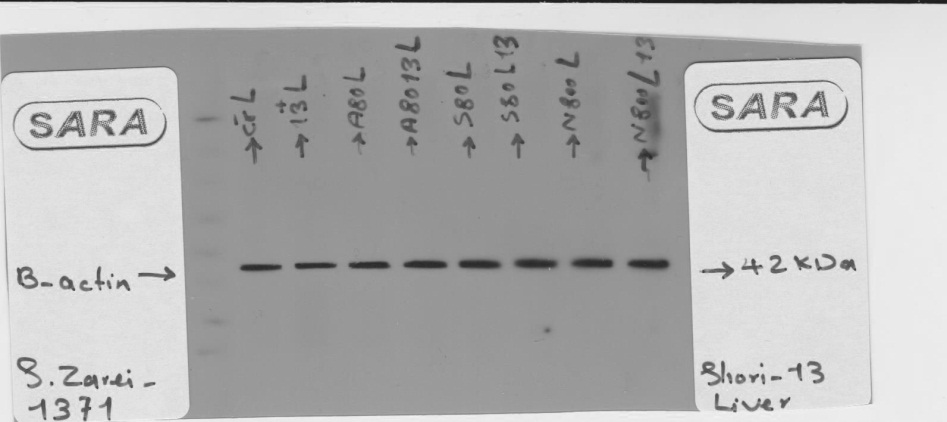
**

**b)**

**
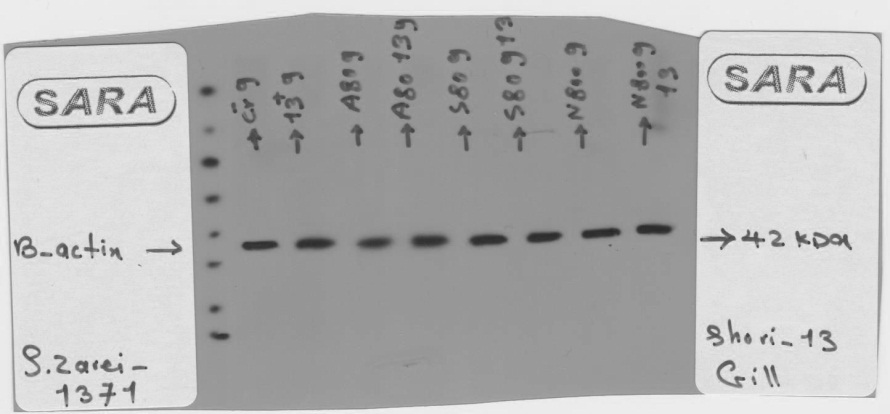
**

**c)**

**
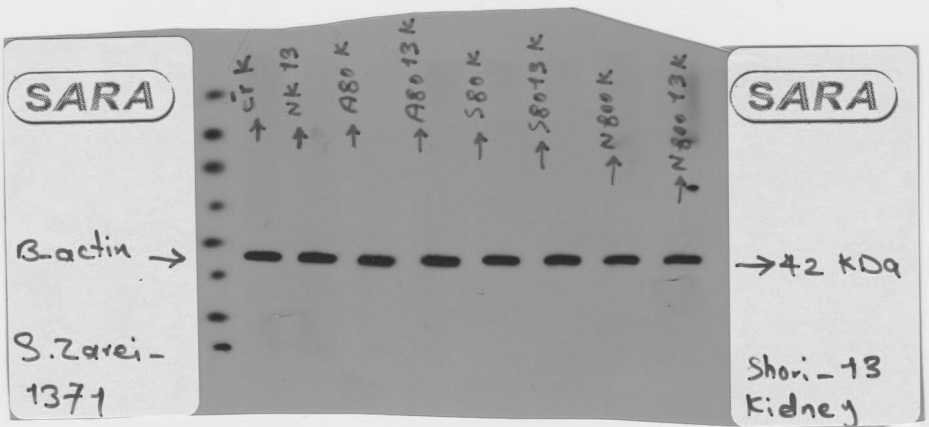
**

**Fig. 8**


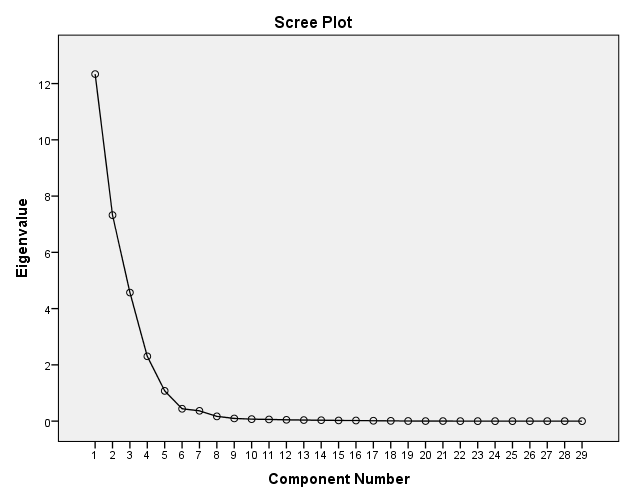


**Tables.**

**Table 1. Analysis of the main components in salinity stress.**

| **Total Variance Explained** | | | | | | | | | |
| --- | --- | --- | --- | --- | --- | --- | --- | --- | --- |
| Component | Initial Eigenvalues | | | Extraction Sums of Squared Loadings | | | Rotation Sums of Squared Loadings | | |
|  | Total | % of Variance | Cumulative % | Total | % of Variance | Cumulative % | Total | % of Variance | Cumulative % |
| 1 | 12.337 | 42.541 | 42.541 | 12.337 | 42.541 | 42.541 | 9.343 | 32.217 | 32.217 |
| 2 | 7.324 | 25.254 | 67.795 | 7.324 | 25.254 | 67.795 | 8.277 | 28.540 | 60.757 |
| 3 | 4.571 | 15.764 | 83.558 | 4.571 | 15.764 | 83.558 | 4.908 | 16.926 | 77.683 |
| 4 | 2.303 | 7.940 | 91.499 | 2.303 | 7.940 | 91.499 | 3.499 | 12.066 | 89.750 |
| 5 | 1.075 | 3.707 | 95.206 | 1.075 | 3.707 | 95.206 | 1.582 | 5.456 | 95.206 |
| 6 | .440 | 1.516 | 96.721 |  |  |  |  |  |  |
| 7 | .366 | 1.261 | 97.982 |  |  |  |  |  |  |
| 8 | .169 | .584 | 98.566 |  |  |  |  |  |  |
| 9 | .095 | .327 | 98.893 |  |  |  |  |  |  |
| 10 | .071 | .246 | 99.138 |  |  |  |  |  |  |
| 11 | .059 | .204 | 99.342 |  |  |  |  |  |  |
| 12 | .047 | .161 | 99.503 |  |  |  |  |  |  |
| 13 | .037 | .126 | 99.629 |  |  |  |  |  |  |
| 14 | .033 | .113 | 99.742 |  |  |  |  |  |  |
| 15 | .025 | .085 | 99.827 |  |  |  |  |  |  |
| 16 | .022 | .076 | 99.903 |  |  |  |  |  |  |
| 17 | .013 | .043 | 99.946 |  |  |  |  |  |  |
| 18 | .008 | .029 | 99.975 |  |  |  |  |  |  |
| 19 | .004 | .013 | 99.988 |  |  |  |  |  |  |
| 20 | .002 | .006 | 99.995 |  |  |  |  |  |  |
| 21 | .001 | .003 | 99.998 |  |  |  |  |  |  |
| 22 | .000 | .001 | 100.000 |  |  |  |  |  |  |
| 23 | .000 | .000 | 100.000 |  |  |  |  |  |  |
| 24 | 3.970E-16 | 1.369E-15 | 100.000 |  |  |  |  |  |  |
| 25 | 2.817E-16 | 9.713E-16 | 100.000 |  |  |  |  |  |  |
| 26 | 7.771E-17 | 2.680E-16 | 100.000 |  |  |  |  |  |  |
| 27 | -1.408E-16 | -4.856E-16 | 100.000 |  |  |  |  |  |  |
| 28 | -2.359E-16 | -8.134E-16 | 100.000 |  |  |  |  |  |  |
| 29 | -3.671E-16 | -1.266E-15 | 100.000 |  |  |  |  |  |  |
| Extraction Method: Principal Component Analysis. | | | | | | | | | |

**Table 2. The scores of each of the variables in the main components.**

| **Component Matrix^a^** | | | | | |
| --- | --- | --- | --- | --- | --- |
| **Treatment** | Component | | | | |
|  | 1 | 2 | 3 | 4 | 5 |
| **Liver- HSP90** | .893 | .344 | .207 | .127 | .047 |
| **Liver- HSP27** | .819 | .368 | .038 | .325 | .144 |
| **GST** | .812 | -.178 | -.181 | -.264 | -.395 |
| **Kidney-HSP70** | .807 | .530 | .136 | .036 | .208 |
| **Kidney-HSP27** | .797 | .500 | .153 | -.282 | .028 |
| **ALP-Liver** | .781 | -.565 | -.054 | .198 | .120 |
| **Gill-HSP70** | .768 | .423 | .381 | -.091 | .205 |
| **Liver-HSP70** | .768 | .510 | .194 | .049 | .164 |
| **Gill-HSP27** | .736 | .553 | .066 | .225 | .186 |
| **AST-Kidney** | .721 | -.606 | .109 | .260 | .102 |
| **Kidney-HSP90** | .709 | .539 | .294 | -.248 | .101 |
| **ALT-Kidney** | .704 | -.625 | .131 | .161 | .156 |
| **AST-Liver** | .700 | -.620 | -.034 | .244 | -.044 |
| **ALP-Kidney** | .692 | -.671 | -.008 | .198 | .133 |
| **Cortisol** | .645 | .433 | -.386 | .206 | -.291 |
| **GPX** | .635 | .513 | .153 | .207 | -.387 |
| **TAC** | .416 | .765 | -.043 | .094 | -.469 |
| **LDH-Kidney** | .646 | -.698 | -.045 | .189 | .058 |
| **ALT-Liver** | .635 | -.672 | -.075 | .192 | -.163 |
| **LDH-Liver** | .579 | -.660 | -.224 | .244 | -.209 |
| **C3** | -.219 | .152 | -.907 | .016 | .272 |
| **Protein Total-Kidney** | -.208 | -.110 | .897 | -.121 | .051 |
| **Protein Total-Liver** | -.469 | -.307 | .791 | -.082 | .044 |
| **LYZ** | .429 | .324 | -.790 | -.204 | .155 |
| **IgM** | .399 | .390 | -.739 | -.259 | .161 |
| **Gill-HSP90** | .610 | .231 | .682 | -.290 | -.043 |
| **Liver Viability (LV)** | -.542 | .498 | .100 | .646 | .102 |
| **Kidney Viability (KV)** | -.577 | .457 | .084 | .645 | .021 |
| **Gill Viability (GV)** | -.551 | .503 | .080 | .634 | -.018 |
| Extraction Method: Principal Component Analysis. a. 5 components extracted. | | | | | |
|  | | | | | |

**Table 3. Correlation assessment between the parameters measured in salinity stress.**

| K-Viability | G-Viability | L-Viability | K-Protein TAM | L-Protein TAM | L-LYZ | L-C3 | L-IgM | L-TAC | L-GPX | L-GST | K-LDH | L-LDH | K-ALT | L-ALT | K-AST | L-AST | K-ALP | L-ALP | L-Cortisol | K-HSP90 | G-HSP90 | L-HSP90 | K-HSP70 | G-HSP70 | L- HSP70 | K-HSP27 | G-HSP27 | L-HSP27 |  |
| --- | --- | --- | --- | --- | --- | --- | --- | --- | --- | --- | --- | --- | --- | --- | --- | --- | --- | --- | --- | --- | --- | --- | --- | --- | --- | --- | --- | --- | --- |
|  |  |  |  |  |  |  |  |  |  |  |  |  |  |  |  |  |  |  |  |  |  |  |  |  |  |  |  | 1 | **L-HSP27** |
|  |  |  |  |  |  |  |  |  |  |  |  |  |  |  |  |  |  |  |  |  |  |  |  |  |  |  | 1 | 0.955^**^ | **G-HSP27** |
|  |  |  |  |  |  |  |  |  |  |  |  |  |  |  |  |  |  |  |  |  |  |  |  |  |  | 1 | 0.811^**^ | 0.735^**^ | **K-HSP27** |
|  |  |  |  |  |  |  |  |  |  |  |  |  |  |  |  |  |  |  |  |  |  |  |  |  | 1 | 0.895^**^ | 0.838^**^ | 0.807^**^ | **L- HSP70** |
|  |  |  |  |  |  |  |  |  |  |  |  |  |  |  |  |  |  |  |  |  |  |  |  | 1 | 0.951^**^ | 0.911^**^ | 0.800^**^ | 0.779^**^ | **G-HSP70** |
|  |  |  |  |  |  |  |  |  |  |  |  |  |  |  |  |  |  |  |  |  |  |  | 1 | 0.923^**^ | 0.941^**^ | 0.927^**^ | 0.956^**^ | 0.905^**^ | **K-HSP70** |
|  |  |  |  |  |  |  |  |  |  |  |  |  |  |  |  |  |  |  |  |  |  | 1 | 0.953^**^ | 0.890^**^ | 0.903^**^ | 0.888^**^ | 0.914^**^ | 0.908^**^ | **L-HSP90** |
|  |  |  |  |  |  |  |  |  |  |  |  |  |  |  |  |  |  |  |  |  | 1 | 0.718^**^ | 0.680^**^ | 0.862^**^ | 0.713^**^ | 0.788^**^ | 0.524^**^ | 0.495^*^ | **G-HSP90** |
|  |  |  |  |  |  |  |  |  |  |  |  |  |  |  |  |  |  |  |  | 1 | 0.810^**^ | 0.876^**^ | 0.923^**^ | 0.895^**^ | 0.849^**^ | 0.965^**^ | 0.839^**^ | 0.724^**^ | **K-HSP90** |
|  |  |  |  |  |  |  |  |  |  |  |  |  |  |  |  |  |  |  | 1 | 0.544^**^ | 0.172^ns^ | 0.689^**^ | 0.656^**^ | 0.435^*^ | 0.600^**^ | 0.627^**^ | 0.690^**^ | 0.661^**^ | **L-Cortisol** |
|  |  |  |  |  |  |  |  |  |  |  |  |  |  |  |  |  |  | 1 | 0.282^ns^ | 0.189^ns^ | 0.248^ns^ | 0.515^*^ | 0.352^ns^ | 0.355^ns^ | 0.345^ns^ | 0.279^ns^ | 0.312^ns^ | 0.499^*^ | **L-ALP** |
|  |  |  |  |  |  |  |  |  |  |  |  |  |  |  |  |  | 1 | 0.988^**^ | 0.153^ns^ | 0.083^ns^ | 0.201^ns^ | 0.407^*^ | 0.233^ns^ | 0.261^ns^ | 0.223^ns^ | 0.159^ns^ | 0.200^ns^ | 0.403^ns^ | **K-ALP** |
|  |  |  |  |  |  |  |  |  |  |  |  |  |  |  |  | 1 | 0.943^**^ | 0.946^**^ | 0.297^ns^ | 0.095^ns^ | 0.188^ns^ | 0.434^*^ | 0.234^ns^ | 0.235^ns^ | 0.237^ns^ | 0.181^ns^ | 0.201^ns^ | 0.390^ns^ | **L-AST** |
|  |  |  |  |  |  |  |  |  |  |  |  |  |  |  | 1 | 0.920^**^ | 0.968^**^ | 0.957^**^ | 0.165^ns^ | 0.150^ns^ | 0.302^ns^ | 0.490^*^ | 0.302^ns^ | 0.342^ns^ | 0.288^ns^ | 0.209^ns^ | 0.282^ns^ | 0.486^*^ | **K-AST** |
|  |  |  |  |  |  |  |  |  |  |  |  |  |  | 1 | 0.882^**^ | 0.917^**^ | 0.899^**^ | 0.900^**^ | 0.239^ns^ | -0.07^ns^ | 0.141^ns^ | 0.341^ns^ | 0.121^ns^ | 0.141^ns^ | 0.156^ns^ | 0.116^ns^ | 0.074^ns^ | 0.272^ns^ | **L-ALT** |
|  |  |  |  |  |  |  |  |  |  |  |  |  | 1 | 0.856^**^ | 0.964^**^ | 0.901^**^ | 0.966^**^ | 0.953^**^ | 0.108^ns^ | 0.158^ns^ | 0.342^ns^ | 0.461^*^ | 0.283^ns^ | 0.360^ns^ | 0.294^ns^ | 0.227^ns^ | 0.220^ns^ | 0.411^*^ | **K-ALT** |
|  |  |  |  |  |  |  |  |  |  |  |  | 1 | 0.776^**^ | 0.891^**^ | 0.823^**^ | 0.890^**^ | 0.855^**^ | 0.852^**^ | 0.312^ns^ | -0.047^ns^ | -0.027^ns^ | 0.283^ns^ | 0.064^ns^ | -0.010^ns^ | 0.023^ns^ | 0.035^ns^ | 0.096^ns^ | 0.273^ns^ | **L-LDH** |
|  |  |  |  |  |  |  |  |  |  |  | 1 | 0.865^**^ | 0.906^**^ | 0.901^**^ | 0.944^**^ | 0.910^**^ | 0.955^**^ | 0.935^**^ | 0.120^ns^ | 0.015^ns^ | 0.141^ns^ | 0.353^ns^ | 0.162^ns^ | 0.174^ns^ | 0.135^ns^ | 0.092^ns^ | 0.154^ns^ | 0.369^ns^ | **K-LDH** |
|  |  |  |  |  |  |  |  |  |  | 1 | 0.568^**^ | 0.626^**^ | 0.579^**^ | 0.677^**^ | 0.563^**^ | 0.639^**^ | 0.590^**^ | 0.662^**^ | 0.565^**^ | 0.431^*^ | 0.433^*^ | 0.563^**^ | 0.436^*^ | 0.446^*^ | 0.467^*^ | 0.595^**^ | 0.317^ns^ | 0.418^*^ | **L-GST** |
|  |  |  |  |  |  |  |  |  | 1 | 0.507^*^ | 0.096^ns^ | 0.072^ns^ | 0.140^ns^ | 0.124^ns^ | 0.206^ns^ | 0.159^ns^ | 0.100^ns^ | 0.197^ns^ | 0.635^**^ | 0.619^**^ | 0.583^**^ | 0.744^**^ | 0.714^**^ | 0.695^**^ | 0.727^**^ | 0.686^**^ | 0.717^**^ | 0.768^**^ | **L-GPX** |
|  |  |  |  |  |  |  |  | 1 | 0.864^**^ | 0.369^ns^ | -0.265^ns^ | -0.135^ns^ | -0.251^ns^ | -0.155^ns^ | -0.192^ns^ | -0.140^ns^ | -0.268^ns^ | -0.142^ns^ | 0.747^**^ | 0.620^**^ | 0.392^ns^ | 0.613^**^ | 0.642^**^ | 0.518^**^ | 0.625^**^ | 0.664^**^ | 0.665^**^ | 0.591^**^ | **L-TAC** |
|  |  |  |  |  |  |  | 1 | 0.395^ns^ | 0.264^ns^ | 0.430^*^ | -0.026^ns^ | 0.010^ns^ | -0.058^ns^ | -0.021^ns^ | -0.079^ns^ | 0.003^ns^ | 0.003^ns^ | 0.113^ns^ | 0.584^**^ | 0.319^ns^ | -0.091^ns^ | 0.287^ns^ | 0.439^*^ | 0.280^ns^ | 0.423^*^ | 0.472^*^ | 0.391^ns^ | 0.362^ns^ | **L-IgM** |
|  |  |  |  |  |  | 1 | 0.698^**^ | -0.059^ns^ | -0.309^ns^ | -0.129^ns^ | -0.199^ns^ | -0.081^ns^ | -0.317^ns^ | -0.198^ns^ | -0.323^ns^ | -0.210^ns^ | -0.206^ns^ | -0.165^ns^ | 0.207^ns^ | -0.323^ns^ | -0.731^**^ | -0.315^ns^ | -0.163^ns^ | -0.385^ns^ | -0.191^ns^ | -0.229^ns^ | -0.105^ns^ | -0.143^ns^ | **L-C3** |
|  |  |  |  |  | 1 | 0.708^**^ | 0.966^**^ | 0.373^ns^ | -0.245^ns^ | 0.430^*^ | 0.071^ns^ | 0.115^ns^ | -0.005^ns^ | 0.053^ns^ | -0.003^ns^ | 0.060^ns^ | 0.069^ns^ | 0.174^ns^ | 0.604^**^ | 0.294^ns^ | -0.138^ns^ | 0.300^ns^ | 0.429^*^ | 0.227^ns^ | 0.363^ns^ | 0.440^*^ | 0.411^*^ | 0.395^ns^ | **L-LYZ** |
|  |  |  |  | 1 | -0.907^**^ | -0.658^**^ | 0.840^**^ | -0.507^*^ | -0.359^ns^ | -0.448^*^ | -0.148^ns^ | -0.280^ns^ | -0.023^ns^ | -0.183^ns^ | -0.080^ns^ | -0.193^ns^ | -0.132^ns^ | -0.245^ns^ | -0.760^**^ | -0.251^ns^ | 0.210^ns^ | -0.379^ns^ | -0.435^*^ | -0.154^ns^ | -0.342^ns^ | -0.384^ns^ | -0.487^*^ | -0.490^*^ | **L-Protein TAM** |
|  |  |  | 1 | 0.810^**^ | -0.801^**^ | -0.728^**^ | -0.739^**^ | -0.232^ns^ | -0.120^ns^ | -0.277^ns^ | -0.118^ns^ | -0.289^ns^ | 0.006^ns^ | -0.133^ns^ | -0.032^ns^ | -0.102^ns^ | -0.088^ns^ | -0.151^ns^ | -0.553^**^ | 0.096^ns^ | 0.478^*^ | -0.048^ns^ | -0.090^ns^ | 0.154^ns^ | -0.010^ns^ | 0.042^ns^ | -0.189^ns^ | -0.246^ns^ | **K-Protein TAM** |
|  |  | 1 | 0.086^ns^ | 0.131^ns^ | -0.268^ns^ | 0.152^ns^ | -0.250^ns^ | 0.163^ns^ | -0.003^ns^ | 0.745^**^ | -0.595^**^ | -0.519^**^ | -0.555^**^ | -0.566^**^ | -0.505^*^ | -0.533^**^ | -0.569^**^ | -0.565^**^ | -0.052^ns^ | -0.229^ns^ | -0.337^ns^ | -0.199^ns^ | -0.114^ns^ | -0.203^ns^ | -0.078^ns^ | -0.338^ns^ | 0.036^ns^ | -0.056^ns^ | **L-Viability** |
|  | 1 | 0.965^**^ | 0.062^ns^ | 0.137^ns^ | -0.265^ns^ | 0.145^ns^ | -0.220^ns^ | 0.213^ns^ | 0.064^ns^ | -0.685^**^ | -0.602^**^ | -0.520^**^ | -0.576^**^ | -0.553^**^ | -0.534^**^ | -0.541^**^ | -0.592^**^ | -0588^**^ | -0.031^ns^ | -0.270^ns^ | -0.337^ns^ | -0.235^ns^ | -0.155^ns^ | -0.220^ns^ | -0.089^ns^ | -0.350^ns^ | -0.013^ns^ | -0.086^ns^ | **G-Viability** |
| 1 | 0.979^**^ | 0.970^**^ | 0.083^ns^ | 0.142^ns^ | -0.281^ns^ | 0.144^ns^ | -0.252^ns^ | 0.161^ns^ | 0.018^ns^ | -0.718^**^ | -0.573^**^ | -0.525^**^ | -0.558^**^ | -0.539^**^ | -0.520^**^ | -0.525^**^ | -0.570^**^ | -0.571^**^ | -0.099^ns^ | -0.315^ns^ | -0.362^ns^ | -0.268^ns^ | -0.192^ns^ | -0.251^ns^ | -0.121^ns^ | -0.395^ns^ | -0.053^ns^ | -0.116^ns^ | **K-Viability** |

**^ns^. Correlation is nonsignificant. ^*^. Correlation is significant at the 0.05 level (2-tailed). ^**^. Correlation is significant at the 0.01 level (2-tailed).**
